# The role of the mitochondrial ribosome in human disease: searching for mutations in 12S mitochondrial rRNA with high disruptive potential

**DOI:** 10.1093/hmg/ddt490

**Published:** 2013-10-02

**Authors:** Paul M. Smith, Joanna L. Elson, Laura C. Greaves, Saskia B. Wortmann, Richard J.T. Rodenburg, Robert N. Lightowlers, Zofia M.A. Chrzanowska-Lightowlers, Robert W. Taylor, Antón Vila-Sanjurjo

**Affiliations:** 1 Institute of Medical Sciences, Ninewells Hospital and Medical School, Dundee University, Dundee DD1 9SY, Scotland, UK,; 2 Wellcome Trust Centre for Mitochondrial Research, Newcastle University, The Medical School, Newcastle upon Tyne NE2 4HH, UK,; 3 Institute of Genetic Medicine, Newcastle University, Newcastle upon Tyne NE1 3BZ, UK,; 4 Centre for Human Metabonomics, North-West University, Potchefstroom, South Africa,; 5 Newcastle University Centre for Brain Ageing and Vitality, Institute for Ageing and Health, Newcastle University, The Medical School, Newcastle upon Tyne NE2 4HH, UK,; 6 Department of Pediatrics and the Institute for Genetic and Metabolic Disease (IGMD), Nijmegen Center for Mitochondrial Disorders (NCMD), Radboud University Medical Center, PO Box 9101, Nijmegen 6500 HB, The Netherlands and; 7 Department of Química Fundamental, Facultade de Ciencias, Universidade da Coruña (UDC), 15071 A Coruña, Spain

## Abstract

Mutations of mitochondrial DNA are linked to many human diseases. Despite the identification of a large number of variants in the mitochondrially encoded rRNA (mt-rRNA) genes, the evidence supporting their pathogenicity is, at best, circumstantial. Establishing the pathogenicity of these variations is of major diagnostic importance. Here, we aim to estimate the disruptive effect of mt-rRNA variations on the function of the mitochondrial ribosome. In the absence of direct biochemical methods to study the effect of mt-rRNA variations, we relied on the universal conservation of the rRNA fold to infer their disruptive potential. Our method, named heterologous inferential analysis or HIA, combines conservational information with functional and structural data obtained from heterologous ribosomal sources. Thus, HIA's predictive power is superior to the traditional reliance on simple conservation indexes. By using HIA, we have been able to evaluate the disruptive potential for a subset of uncharacterized 12S mt-rRNA variations. Our analysis revealed the existence of variations in the rRNA component of the human mitoribosome with different degrees of disruptive power. In cases where sufficient information regarding the genetic and pathological manifestation of the mitochondrial phenotype is available, HIA data can be used to predict the pathogenicity of mt-rRNA mutations. In other cases, HIA analysis will allow the prioritization of variants for additional investigation. Eventually, HIA-inspired analysis of potentially pathogenic mt-rRNA variations, in the context of a scoring system specifically designed for these variants, could lead to a powerful diagnostic tool.

## INTRODUCTION

Currently, more than 550 mitochondrial DNA (mtDNA) mutations and sequence variants have been associated with human disease (1). Since it has been estimated that the minimal prevalence of clinically manifesting mtDNA disease in adults is ∼1 in 10 000 (2), establishing whether mtDNA mutations are pathogenic is of major importance for genetic counseling and patient support. Whilst important, the assignment of pathogenicity to mtDNA mutations is a difficult task. This is in no small part due to high levels of mtDNA variation in human populations and the extremely heterogeneous clinical presentations of many known mtDNA mutations. Due to these and other difficulties, the evidence for pathogenicity has been rather weak in some cases, especially in the context of rRNA variation. In fact, some authors have suggested that a number of these associations might be erroneous (3–7).

DiMauro and Schon (8) were the first to describe criteria for the identification of mutations in mtDNA linked to disease. Since then, our understanding of the elements associated with pathogenicity has substantially changed. For example, the first element cited by DiMauro and Schon for the identification of pathogenic mutations was that the variant must be heteroplasmic (i.e. the mutated and wild-type variants co-existing in the cells of the affected tissues). More recently, it has become appreciated that certain pathogenic variants may exist as homoplasmic mutations with variable degrees of penetrance (9). These changes in our appreciation of the elements required for pathogenicity, together with the fact that the data available in databases has grown enormously, makes it imperative to find a new framework for the analysis of mitochondrially encoded rRNA (mt-rRNA) variations. The emergence of scoring systems has provided a much needed tool for the evaluation of certain categories of mtDNA mutations and should provide guidance to future research on mt-rRNA variation (6,7,10–12).

With all probability, an important subset of pathogenic mtDNA variations map to the mt-rRNA genes; however, they remain the most difficult genes in which to confirm the pathogenicity of an mtDNA variant. Since the mitochondrial ribosome (mitoribosome) translates all 13 proteins encoded by the mitochondrial genome, mutations leading to ribosome dysfunction should result in a respiratory chain defect and, therefore, cause mitochondrial disease. This prediction was confirmed by the identification of alterations in the nuclear and mitochondrial genome that affect the mitochondrial translational machinery. For example, a small number of highly deleterious mutations have been identified in the nuclear genes encoding protein components of mitoribosomes, mitochondrial translation factors and elements of the apparatus involved in the maturation of mitoribosomes (13–20). The severity of these mutations clearly indicates that interfering with mitochondrial protein synthesis can lead to highly deleterious phenotypes. Not surprisingly, deleterious mutations have also been identified in the rRNA component of the mitoribosome. Two such mutations, 908A>G (m.1555A>G) and 847C>U (m.1494C>T), are well-known causes of hearing loss (21). In addition, there is a large list of other mt-rRNA mutations whose pathogenic role is yet to be convincingly proven.

Thus, despite the identification of base substitutions in the mt-rRNA of patients, the evidence supporting the pathogenicity of mt-rRNA variations is, at best, circumstantial, except for the two cases mentioned above. The reasons for this are many. First, ascertaining the magnitude of the actual biochemical defect caused by such variations is often complicated by incomplete penetrance, as well as by the possible existence of nuclear modifiers. Secondly, the existence of numerous human haplogroups further complicates the correct assignment of mutations, as the penetrance of some variants might change in different genetic contexts (22). Thirdly, the intractability of the mitochondrial translation apparatus and the lack of methods for the manipulation of the mitochondrial genome make it impossible to test the effect of these mutations directly. Finally, all of this is complicated by the lack of agreement among authors on the criteria that should be used to evaluate pathogenicity in the context of mt-rRNA mutations (5,23). For all these reasons, it is imperative that a robust system is devised that allows the assessment of the biochemical defect caused by mt-rRNA mutations.

Scoring systems have been developed and validated for the analysis of the potential pathogenicity of both mt-tRNA and mitochondrially encoded complex I subunits (6,7,10–12). The existence of a substantial number of known mutations and gold-standard experimental procedures, including biochemical and *trans*mitochondrial-cybrid evidence, were important in developing these scoring systems. Unfortunately, these elements are largely lacking in the case of mt-rRNA variations. To fill this void, we propose taking advantage of the universal conservation of the rRNA structure (24) in order to assess the disruptive potential of mt-rRNA variations. Our approach, which we have named heterologous inferential analysis (HIA) predicts the structural and/or functional role of a particular mt-rRNA residue by extrapolating conclusions drawn from evidence pertaining to its structurally equivalent position in a heterologous ribosome. HIA is used in this paper to analyze a subset of extremely rare variations mapping to the small ribosomal subunit (SSU) of the mitoribosome, which are suspected to be associated with a respiratory chain defect. Our goal is to establish whether these mt-rRNA variations have a potentially disruptive effect on the function of the mitoribosome. While HIA alone cannot, in most cases, be used to establish pathogenicity beyond doubt, we believe that it constitutes an important first step toward building a scoring system with clinical relevance that can be used to predict the pathogenic potential of mt-rRNA variations. At the same time, HIA's predictions will allow the prioritization of variants for additional functional investigation. Additionally, for certain mt-rRNA mutations for which sufficient information regarding the genetic and pathological manifestation of the mutations are available, HIA data can be used to predict their pathogenicity.

## RESULTS

Our search through the literature for mt-12S rRNA variations possibly associated with disruption of mitochondrial function, as defined in the section Materials and Methods, resulted in the identification of 161 different variations, corresponding to 152 different sites, almost 16% of the molecule (not shown). Evaluation of evidence used to identify mtDNA mutations in other mtDNA genes indicates that the absence of the variant in controls is important evidence of pathogenicity. In the absence of proper experimental controls in most of the cases studied here, we turned to the GenBank and Phylotree databases to screen for extremely rare variations, i.e. variations with zero appearances in these databases. While we know this criteria would excluded the known pathogenic variants, 1555A>G with 29 appearances and 1494C>T with three appearances, it was hoped that such a criterion would identify a group of variants most likely to yield pathogenic or potentially pathogenic mutations.

### Correlation between phylogenetic conservation and the distribution of variations in mt-12S rRNA

**Figure 1.**
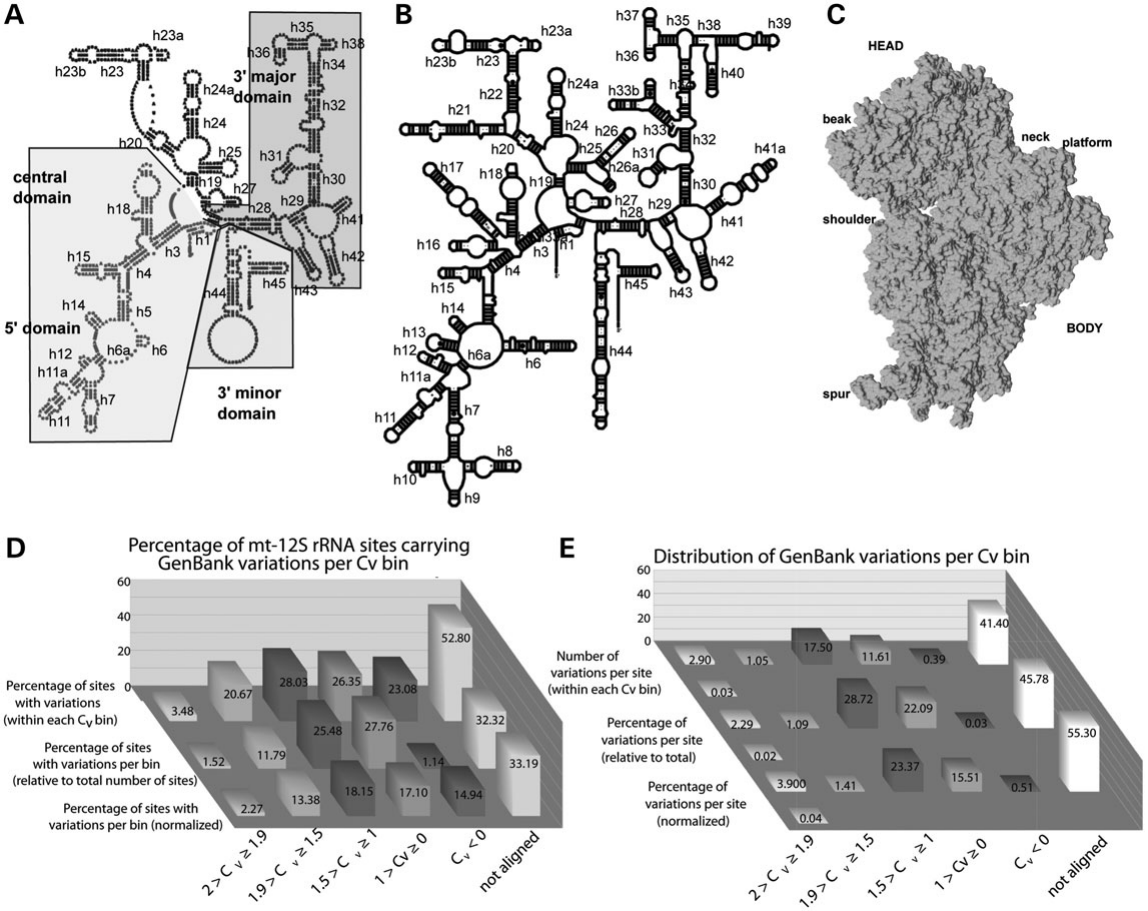
(**A** and **B**) SSU secondary-structure maps. The secondary-structure map of the human mt-12S rRNA is shown in (A). Helices numbered according to the *E. coli* secondary structure model, shown in B. (**C**) Three-dimensional structure of the *E. coli* SSU with landmarks indicated. (**D**) Percentage of mt-12S rRNA positions carrying GenBank variations within each C_v_ bin. C_v_ bins were defined as described at the Comparative RNA Web (CRW) Site (25,26). *Back row*: per-bin percentage of sites with GenBank variations, relative to the total number of residues in each C_v_ bin. *Middle row*: per-bin percentage of sites with GenBank variations, relative to the total number of mt-12S rRNA sites with variations present in GenBank. *Front row*: normalized, per-bin percentage of sites with GenBank variations, relative to the normalized total number of mt-12S rRNA sites with such variations (normalization is to the C_v_ bin with the highest number of sites, i.e. 1 > C_v_ ≥ 0; the normalized values used for these calculations are shown in Table 1). *X axis*: C_v_ bins. *Y axis*: percentage value. (**E**) Distribution of mt-12S rRNA GenBank variations per C_v_ bin. The distribution is shown as the number of GenBank variations per site within each C_v_ bin (two back rows, see main text for calculation details); as the per-bin percentage of variations, relative to the total number of mt-12S rRNA variations present in GenBank (two middle rows); and as the per-bin, normalized percentage of variations, relative to the normalized total number of mt-12S rRNA variations with such variations (normalization as in D). *Odd rows*: as adjacent even row but corrected after discarding the outlier residue 180A (m.827A) (see main text. Only shown for most conserved bin). *X axis*: C_v_ bins. *Y axis*: number of variations per site (two back rows), percentage value (four front rows).

The accuracy of phylogeny-driven algorithms for the prediction of rRNA structure has been amply demonstrated (24). Therefore, we reasoned that the universal conservation of the rRNA fold must be central to any inferential attempt to ascertain the potentially disruptive effect of mutations in mt-rRNA. Figure 1A shows the secondary structure map of the human 12S rRNA with all domains and helices numbered according to the nomenclature used for the bacterial 16S rRNA, shown in Figure 1B (25). Figure 1C shows the three dimensional structure of the *Escherichia* *coli* SSU with landmarks indicated (accession code 2I2P). Despite the loss of many peripheral RNA segments in the human 12S rRNA, the overall fold is clearly conserved (Fig. 1A and B), thus allowing the identification of equivalent residues in both structures. Only for positions lying in areas of little structural homology does the identification of heterologous structural equivalents become impossible.

The aforementioned idea that rare variations might be good candidates for pathogenic mutations would require a non-random distribution of variations in the *MT-RNR1*gene, i.e. that an inverse correlation exists between phylogenetic conservation and the number of variations per residue. However, our analysis of near 18 000 complete sequences of the human mt-genome currently deposited in GenBank showed a high prevalence of mt-12S rRNA sites carrying variations, i.e. 27.6% of all sites (Table 1). This prompted us to consider whether such a correlation between phylogenetic conservation and the distribution of variations per mt-12S rRNA residue actually exists. Conservation values C_v_s for SSU rRNA residues have been calculated by using 8513 SSU rRNA sequences from all three phylogenetic domains, Eubacteria, Archaea and Eucarya (including nuclear, mitochondria and chloroplast sequences) (25). According to the authors, 100% conservation would yield a C_v_ of 2, 90% conservation would result in C_v_s around 1.5, 60–80% conservation in C_v_s near 1.0, 40–50% conservation in values approaching 0.5 and total lack of conservation in values below 0 (26). Since C_v_s constitute measurements of universal rRNA conservation and, thus, should have relevance in all ribosomal systems, including organellar ones, one would expect that the number of variations at every position should correlate with their C_v_. Accordingly, positions with higher C_v_s, i.e. more highly conserved, would display a lower number of variations than positions with lower C_v_s. To test this hypothesis we aligned the 954 residues of mt-12S rRNA and the 1542 residues of *E. coli* 16S rRNA by using the secondary-structure maps as a reference (not shown). As a source of mt-12S rRNA variations we used the GenBank database. After extracting all the variation data for every mt-12S rRNA position, we compared these data to the calculated C_v_s for the same position.

**Table 1.** Distribution of GenBank variations within conservation bins

Conservation value (C_v_)	Number of sites per category	Number of sites carrying variations per category	Normalized number of sites carrying variations per category	Number of variations per category	Normalized number of variations per category
2 > C_V_ ≥ 1.9	115	4 (3)^a^	9.72	333 (3)^a^	809.13 (7.29)^a^
1.9 > C_v_ ≥ 1.5	150	31	57.25	158.00	291.77
1.5 > C_v_ ≥ 1	239	67	77.65	4182.00	4846.92
1 > C_v_ ≥ 0	277	73	73.00	3216.00	3216.00
C_v_ < 0	13	3	63.92	5.00	106.54
Not aligned	161	85	146.24	6666.00	11 468.83
Totals	955	263	427.78	14 560.00	20 739.20

Corrected values after subtraction of values corresponding to outlier residues shown in brackets. Normalization is to the bin with the highest number of sites carrying variations (1 > C_v_ ≥ 0). In brackets are the values obtained for the most conserved bin (2 > C_v_ ≥ 1.9) after neglecting the 330 appearances of the outlier residue 180A (m.827A).

^a^Values calculated for the 2 > C_V_ ≥ 1.9 category after neglecting the 330 appearances at position 827.

When the percentage of mt-12S rRNA positions carrying GenBank variations in every C_v_ bin is plotted, a clear picture emerges. Only 4 of 115 virtually universally conserved residues carried variations, corresponding to 3.48% of all such sites (Table 1 and Fig. 1D, back row). The percentage of sites carrying variations increases sharply as we move into less conserved categories. By definition, the category of residues with C_v_s < 0 is not well represented within mt-12S rRNA (with only 13 aligned residues in this bin). Thus, this bin must be considered as a non-significant element of the analysis. Instead, 161 non-conserved mt-12S rRNA residues were placed in an additional category, named ‘not aligned’ and the associated percentage of variations was calculated. Strikingly, almost 53% of the residues in this category carried variations, an observation that is in clear agreement with our hypothesis of a non-random distribution of variations in human mtDNA. Also shown in Figure 1D is the percentage of sites with variations within each bin, relative to the total number of such sites in the whole mt-12S rRNA (middle row). Additionally, to account for the different bin sizes, we normalized the number of sites with variations in every bin to the bin with the highest number of such sites, namely the 1 > C_v_ ≥ 0 bin (Table 1). These data were used to calculate a normalized percentage of sites with variations per bin (front row of Fig. 1D). Again, the normalized data clearly follows a trend that is consistent with our hypothesis, i.e. the existence of an inverse correlation exists between phylogenetic conservation and the number of variations per residue. The trend is particularly strong for the bins with highest (2 > C_v_ ≥ 1.9 and 1.9 > C_v_ ≥ 1.5) and lowest (‘not aligned’) C_v_s values.

We also investigated the correlation between the total number of variations recorded in GenBank (all possible variations per site were taken into account for this purpose) and C_v_ values. This was done by dividing the total number of variations recorded at all sites within a C_v_ bin by the number of sites in the bin (Fig. 1E, rows 5 and 6). The two most conserved categories clearly display the lowest numbers of variations per site, i.e. 2.90 variations/site for the most conserved bin (2>C_v_ ≥ 1.9) and 1.05 variations/site for the next bin (1.9 > C_v_ ≥ 1.5) (Fig. 1E, 6th row). Notably, a single residue from the nearly invariant bin (2 > C_v_ ≥ 1.9), namely position A827, accounts for 330 out of the 333 variations in the bin (Table 1). When this outlier is excluded from the calculations, the number of variations per site for the nearly invariant bin becomes 0.03 (Fig. 1E, 5th row from the front), in agreement with the extremely high conservation of the residues in this bin. Similarly to Figure 1D, the number of variations per site increases sharply as we move into the less conserved bins, reaching a maximum at the ‘not-aligned’ bin (41.40 variations/site). When the total number of variations per bin is plotted as a percentage of the total number of mt-12S rRNA variations recorded in GenBank, the two most conserved bins account for only 3.38% of the total number of variations (1.11% if the aforementioned outlier is excluded). On the contrary, the ‘not-aligned’ bin accounts for >45% of the variations (Fig. 1E, 3rd and 4th rows). A similar trend is observed after normalization of the percentages to account for bin size (Fig. 1E, 1st and 2nd rows). Again, the data shown in Figure 1E are in agreement with our hypothesis.

In summary, our comparison of phylogenetically derived data on SSU rRNA conservation and the human mtDNA records currently available in GenBank clearly shows that the distribution of variations in mt-12S rRNA is non-random. This distribution shows a strong inverse correlation with the phylogenetically derived conservation value, C_v_. In addition to confirming the strength of comparative methods for the study of rRNA structure, these results validate the use of the GenBank and Phylotree databases as criteria for the identification of extremely rare variations. Doing this, one would expect that an important proportion of such variations could map to highly conserved residues with large disruptive potentials and therefore, have important roles in the function of the mitoribosome.

For all the reasons evidenced above, a group of very rare mt-12S rRNA variants were examined in this study. Specifically, we have focused on 52 variations, including 46 mutations with zero appearances in published controls and in the MITOMAP, GenBank and Phylotree databases, plus the m.896A>G variant, included due to their relevance to the presented results (Table 2 and Supplementary Material, Table S1). We have also analyzed the following five variants: the ‘proven’ pathogenic mutations 847C>U (m.1494C>T) and 908A>G (m.1555A>G), included as HIA positive controls (Table 2); the two common population changes with an abundance over 5% of the GenBank population, 791A>G (m.1438A>G) and 62G>A (m.709G>A), included as negative HIA controls ( Supplementary Material) and the aforementioned, highly conserved outlier 180A>G (m.827A>G), included to test whether a rationale to its strange behavior could be discovered (Supplementary Material).

**Table 2.** Classification of mt-SSU rRNA mutations according to their predicted disruptive potential on ribosomal function

Disruptive power	*E. coli* equivalent	Conservation of position according to CRW^a^	Ci_Univ_	Ci_1ry_	Ci_2ry_	Ci_Tot_	
Unlikely							
249A>G (m.896A>G)	G524	g	3	4	5	19	
		Average	NA	NA	NA	19	
		SD	NA	NA	NA	NA	
Undetermined							
67A>U (m.714A>T)	G112	g	3	3	5	17	
90C>U (m.737C>T)	Unknown	?	NI	NI	NI	NI	
103G>C(m.750G>C)	Unknown	?	NI	NI	NI	NI	
125A>G (m.772A>G)	C271^b^	x	0	2	2	6	
167A>G(m.814A>G)	G351	o	2	4	4	16	
282A>U (m.929A>T)	G557	x	0	0	3	3	
296G>A (m.943G>A)	G577	g	3	3	5	17	
308A>G (m.955A>G)	Unknown	?	NI	NI	NI	NI	
313C>A(m.960C>A)	Unknown	?	NI	NI	NI	NI	
314U>A(m.961T>A)	Unknown	?	NI	NI	NI	NI	
314InsC(5)(m.961InsC(5))	Unknown	?	NI	NI	NI	NI	
314delU(m.961delT)	Unknown	?	NI	NI	NI	NI	
361A>U (m.1008A>T)	G711	w	1	2	4	10	
364C>A(m.1011C>A)	G714	g	3	2	4	14	
474A>G (m.1121A>G)	Unknown	?	NI	NI	NI	NI	
485U>C (m.1132T>C)	C879	w	1	4	4.5	14.5	
878C>G(m.1525C>G)	Unknown	?	NI	NI	NI	NI	
888U>C(m.1535T>C)	Unknown	?	NI	NI	NI	NI	
		Average	1.71	2.63	3.63	8.69	Average
		SD	0.98	0.90	0.90	0.92	SD
NEE							
40G>A(m.687G>A)	G46	w	1	3	5	13	
257C>U(m.904C>T)	A532	o	2	4	5	17	
265U>A(m.912T>A)	G540	w	1	1	4	8	
277A>U (m.924A>T)	U552	w	1	1	4	8	
620U>A (m.1267T>A)	U1062	u	3	3	4.5	16.5	
642G>C (m.1289G>C)	U1083	o	2	2	5	13	
698G>A (m.1345G>A)	G1215	o	2	3	5	15	
701G>A (m.1348G>A)	G1218	w	1	2	5	11	
727A>G (m.1374A>G)	C1245	w	1	1	2.5	6.5	
733G>A (m.1380G>A)	G1294	w	1	1	4.5	8.5	
742G>A (m.1389G>A)	G1304	g	3	2	5	15	
897A>U (m.1544A>T)	C1479	x	0	3	4	10	
929G>A (m.1576G>A)	G1511	o	2	4	5	17	
939G>A(m.1586G>A)	C1521	w	1	4	5	15	
		Average	1.38	1.79	4.34	11.13	Average
		SD	0.78	1.19	0.74	3.83	SD
Likely							
450G>A (m.1097G>A)	G809	o	2	4	5	17	
507A>C (m.1154A>C)	A901	a	3	3	5	17	
676G>A (m.1323G>A)	G1193	o	2	4	4.5	16.5	
680G>A (m.1327G>A)	A1197	w	1	3	5	13	
852U>C (m.1499T>C)	G1415	w	1	1	4.5	8.5	
		Average	1.50	2.31	4.79	13.41	Average
		SD	0.84	1.22	0.27	3.70	SD
Expectedly							
232U>C(m.879T>C)	C507	c	3	3	5	17	
255G>A (m.902G>A)	G530	G	4	5	5	23	
522G>A (m.1169G>A)	U916	o	2	5	5	19	
533U>G (m.T1180T>G)	G927	w	1	4	5	15	
579C>G (m.1226C>G)	C972	C	4	5	5	23	
580G>A (m.1227G>A)	G973	g	3	4	5	19	
910A>C (m.A1557A>C)	A1492	A	4	4	4	20	
915G>A (m.1562G>A)	G1497	G	4	6	5	25	
919C>U (m.1566C>T)	C1501	c	3	5	5	21	
		Average	2.57	4.39	4.86	19.77	Average
		SD	1.05	0.88	0.33	3.15	SD
Proven							
847C>U (m.1494C>T)	A1410	w	1	4	4	14	
908A>G (m.1555A>G)	U1490	w	1	3	4	12	
		Average	1.89	2.99	1.89	11.90	Average
		SD	1.31	1.45	1.50	6.71	SD

NI, no information; NA, not applicable. The table includes all mitochondrial mutations meeting the criteria described in the section Materials and Methods. Only the mutations within the ‘likely’ and ‘expectedly’ categories have been analyzed in detail in this paper. Cis were calculated as described in the section Materials and Methods. Whenever possible, Cis were averaged in a per-category fashion and a standard deviation was calculated.

^a^Cannone *et al.* (25) nomenclature for primary standard conservation across all three phylogenetic kingdoms is included. A, C, G, U, N, nucleotide is conserved in 98–100% of the sequences in the alignment; a, c, g, u, n, nucleotides is conserved in 90–100% of the sequences in the alignment; o, nucleotide is conserved in 80–90% of the sequences in the alignment; w, nucleotide is conserved in <80% of the sequences in the alignment; x, position is conserved in <95% of the sequences in the alignment.

^b^Tentative assignment.

### Conservation index (Ci) analysis

Many authors have relied heavily on very simple Cis for assessing the potential pathogenicity of mt-rRNA mutations. We have calculated three different types of Cis: two based on primary structure conservation, Ci_Univ_ and Ci_1ry_; one based on secondary structure conservation, Ci_2ry_; and a composite Ci which combined the information obtained from the other three, Ci_Tot_. The results are displayed in Table 2. A quick glance of the table clearly shows the failure of Cis to predict pathogenicity. For example, the two known pathogenic mutations 847C>U (m.1494C>T) and 908A>G (m.1555A>G) display rather low Cis. The implications of this are discussed below.

### HIA analysis

The results of HIA analysis are shown in Table 2. Out of the 49 mutations analyzed (the negative controls and outlier were not included in the table), we found 1 whose disruptive potential on the function of mitoribosomes is ‘unlikely disruptive’, 18 which cannot be classified due to local structural differences between the mitochondrial and the heterologous ribosomes (‘undetermined’), 14 which are regarded as ‘not enough evidence (NEE)’, 5 which are ‘likely disruptive’ and 9 which are ‘expectedly disruptive’ (see Materials and Methods). Finally, the pathogenic mutations 847C>U (m.1494C>T) and 908A>G (m.1555A>G) were classified within the ‘proven’ category.

**Figure 2.**
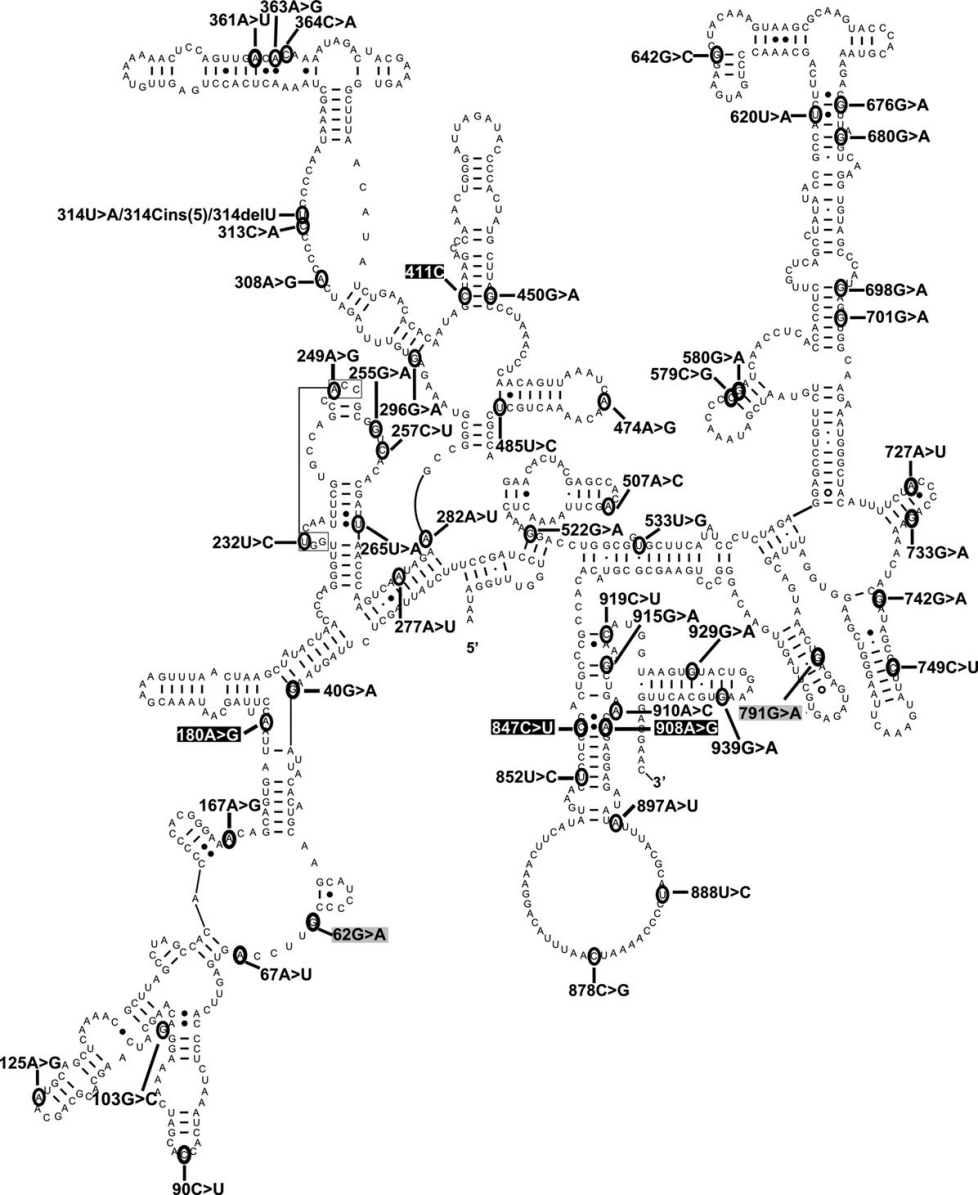
Secondary structure map of the human 12S mt-rRNA showing sites of mutation. The secondary-structure map of human mt-12S rRNA was obtained from the CRW site (25). Sites of mutation analyzed in the main text are shown in black font. Other important sites described in the text (including HIA positive controls) are shown in white font over the black background. HIA negative controls are shown in black font over the gray background.

**Figure 3.**
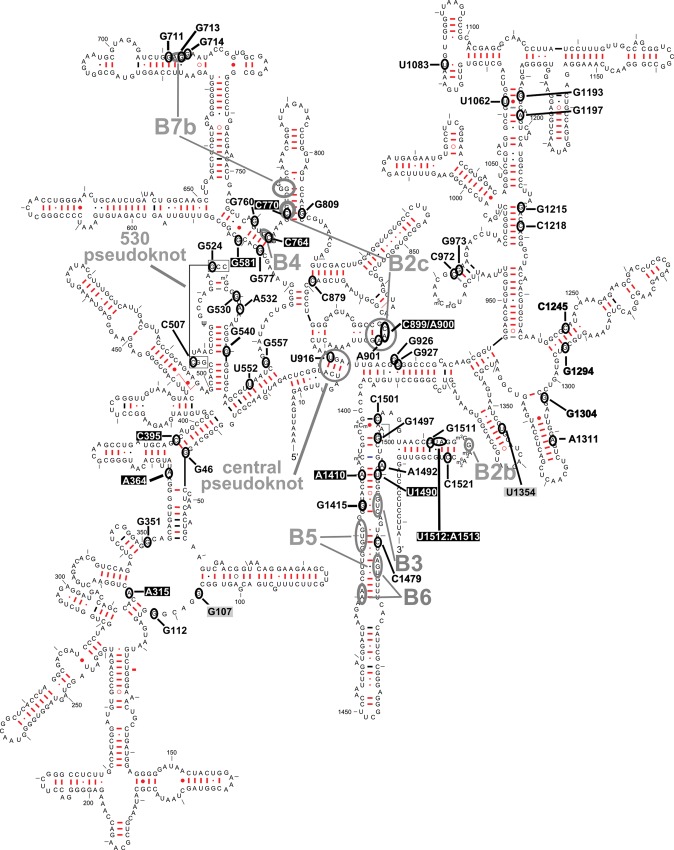
Bacterial equivalents of mitochondrial mutations. Bacterial equivalents of analyzed mutations are shown in black font on the secondary-structure map of *E. coli* 16S rRNA (25). Other important sites described in the text (including HIA positive controls) are shown in white font over the black background. HIA negative controls are shown in black font over the gray background.

**Figure 4.**
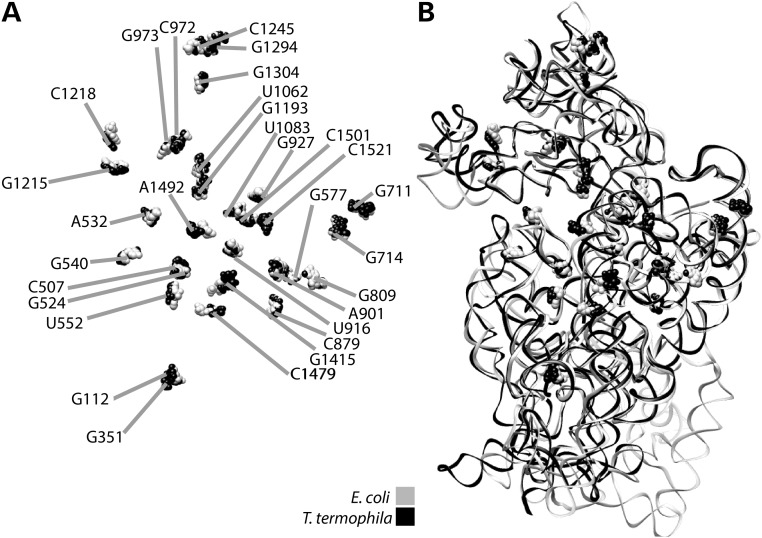
Placement of heterologous equivalents of mitochondrial mutations on the high-resolution structures of *T. thermophila* and *E. coli*. (**A**) Ball representation of superposed heterologous residues. *T. thermophila*, grey; *E. coli*, black. *E. coli* numbering indicated. (**B**) Same residues onto ribbon models of the *T. thermophila* and *E. coli* SSUs. RCSB accession codes 2I2P and 2XZM.

Figure 2 shows the location of the mutations with the highest disruptive potential, namely the ‘likely’ and ‘expectedly disruptive’ categories, on the secondary-structure map of human mt-12S rRNA. The location of the equivalent residues in *E. coli* is shown in Figure 3. While the predictive power of phylogenetically derived secondary structure maps has been amply confirmed experimentally by high-resolution structures of ribosomes from all three domains of life (24,27–29), we sought to confirm the accuracy of our assignment of heterologous residues by independently performing such an assignment in two SSU rRNAs from distantly related organisms. This was followed by their visualization in superposed high-resolution structures. To this end, we used phylogenetically derived secondary-structure maps and high-resolution crystal structures of SSUs from *Tetrahymena thermophila* (Eucarya) and *E. coli* (Eubacteria), both organisms more distant phylogenetically than eubacteria and mitochondria. The results of this comparison are displayed superposed onto the models of SSU rRNAs from these two organisms (Fig. 4 and Supplementary Material, Movie S1). Our results show a perfect structural match for all the studied mitochondrial positions as long as structurally equivalent residues can be located in phylogenetically derived, secondary structure maps of the heterologous ribosomes. The structural agreement between the predicted positions in both SSUs demonstrates that equivalence at the secondary structure level translates into accurate superposition in 3D. Thus, these data demonstrate the validity of comparative analysis to accurately predict the location of such structural equivalents in heterologous 3D ribosomal structures. Structural placement of mitochondrial mutations in heterologous ribosome structures provides the basis for HIA.

Below, we briefly describe the results of the HIA analysis for the mutations arranged within the ‘likely’ and ‘expectedly disruptive’ categories.

### Bridge B2c

**Figure 5.**
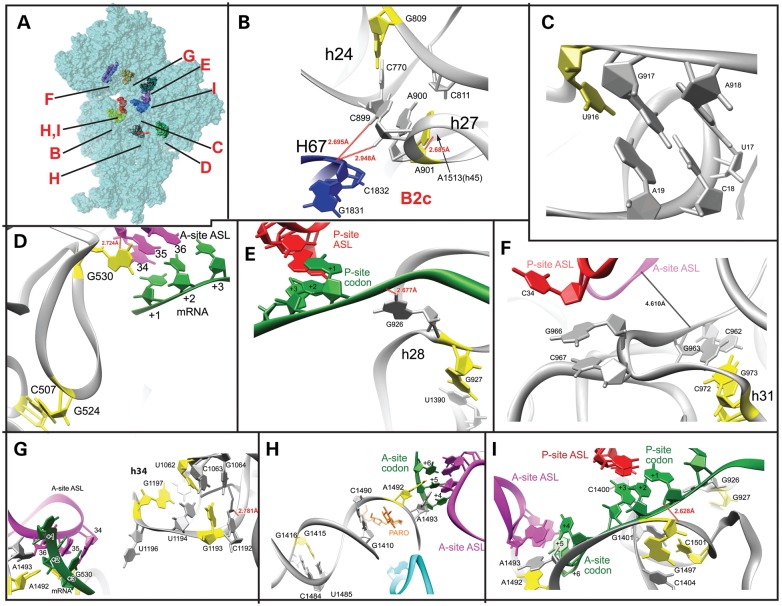
Placement of mitochondrial mutations in the structures of bacterial SSUs. (**A**) Surface representation of the *E. coli SSU* showing the location of the residues shown in B–I. (**B**) *530* pseudoknot. RCSB accession code 2J00. (**C**) Central pseudoknot. RCSB accession code 2I2P. (**D**) Bridge b-B2c. RCSB accession codes 2I2P and 2I2T. (**E**) *927* region. RCSB accession code 2J02. (**F**) *972* region. RCSB accession code 2J02. (**G**) Mutations in *b-h34* and A-site codon–anticodon interaction. RCSB accession code 2J00. (**H**) *b-h44*. The aminoglycoside antibiotic paromomycin is shown in orange. RCSB accession code 2J00. (**I**) Decoding region of *b-h44*. RCSB accession code 2J00. (B–I) Bacterial equivalents of mitochondrial mutations shown in yellow. Other residues mentioned in the main text are shown in gray. 16S rRNA shown in gray. P-site ASL in red, A-site ASL in purple, mRNA in green, ribosomal proteins in black and 23S rRNA in blue. Inter-atomic distances are indicated, but note that ribbon representations do not necessarily reflect accurate interatomic distances involving backbone atoms.

Ribosomal bridge mt-B2c has the same chemical composition in human mitochondria and in bacteria (30). Bridge b-B2c is formed in *E. coli* by the packing of residues *770:771* of b-h24, *899:901* of b-h27 and *1512:1513* of b-h45, against the LSU residues *1831:1833* of b-H67 (Fig. 3) (29,31,32). The mutation 450G>A (m.1097G>A), which was identified in a COX-deficient colonic crypt, maps to this region of 12S rRNA (33) (Figs 2 and 3). The equivalent position in *E. coli*, *G809*, is base paired to b-B2c residue *C770* (Figs 3 and 5B). The evidence for the possible involvement of the 450G (m.1097G) in the incorrect functioning of bridge mt-B2c appears quite likely, since the disruption of the *G809:C770* base pair by mutagenesis has been shown to be detrimental to ribosomal function (34,35). In addition, mutations disrupting Watson:Crick geometry at the adjacent *G769:C810* base pair were highly deleterious (36,37). In the specific case of the 450G>A (m.1097G>A) mutation, the resulting C·A mismatch between 411C and 450G could adopt wobble geometry and thus, impose a rather small structural distortion at the base of m-h24. Indeed, such a configuration has been observed in ∼3.1% of mitochondrial SSU rRNAs (25). Further evidence is necessary to establish whether such geometry would be tolerated in this important region of the human mitoribosome.

One more variation in the neighborhood of mt-B2c, an A>C transition at position 507 (m.1154A>C), was identified in the 12S rRNA of a patient with hearing loss (38). The *E. coli* equivalent to this residue is *A901*, located in the highly conserved tetraloop that caps b-h27 (Fig. 5B). In the *E. coli* ribosome *A901* is involved in tertiary contacts with the ribose of residue *811C* at the base of b-h24, and with the backbone of residue *A1513* in b-h45 (39), underscoring its important role in the stabilization of the complicated structure of the region. The importance of base identity in the tetraloop, as well as that of the correct packing of b-h27 and b-h24 for ribosomal function has been demonstrated in mutagenesis studies (40). In particular, variations at *A901* resulted in less than half of the normal ribosome activity and moderate misreading effects (40).

In summary, the functioning of bridge mt-B2c could be compromised by two mutations found in human 12S rRNA, 450G>A (m.1097G>A) and 507A>C (m.1154A>C) (33,38). Mutagenesis studies performed in bacteria around the heterologous equivalent of 450G (m.1097G) indicated an important role of this region in ribosomal function. However, it is still possible that the helical geometry introduced by the G>A mutation in the organellar ribosome could be accommodated without a major deleterious effect. As for 507A>C (m.1154A>C), the paternal inheritance of the deafness pattern rules out a primary causative effect for the mutation (38). However, the disruptive potential of the base change is quite high, in agreement with the possibility that 507A>C (m.1154A>C) might contribute to the hearing defect. As a result, we are prompted to assign both mutations to the ‘likely disruptive’ category.

### Central pseudoknot

A G to A mutation at position 522 (m.1169G>A) in the central pseudoknot of the SSU has been found in a patient with renal carcinoma (41). Figure 5C shows position *U916*, the *E. coli* equivalent to 522G (m.1169G) that is base paired to residue *A19* within the central pseudoknot (Figs 3 and 5C). This universally conserved structure, whose formation is believed to constitute a crucial step during the maturation of the SSU (42), has been thoroughly studied by mutagenesis in bacteria. The results were clear. Essentially, base complementarity and not primary sequence were required to maintain ribosomal function (43–45). Thus, it appears reasonable to assume that the 522G>A (m.1169G>A) mutation should result in a defective SSU and so we have assigned this mutation to the ‘expectedly disruptive’ category.

### The 530 loop

The 530 loop is one of the most important functional centers of the ribosome and, as such, its primary sequence is highly conserved phylogenetically across all three kingdoms of life (Figure 5). A homoplasmic G to A mutation was identified at position 255 (m.902) in a COX-deficient sample of urothelial cell patches (46). The bacterial equivalent is key residue *G530* from which the loop receives its name. *G530* has been shown to participate in monitoring the fidelity of decoding by checking the codon-anti-codon interaction at the A site (Fig. 5D and G) (47,48). Since alterations at *G530* have been shown to result in dominant lethal phenotypes (49), we must include the 255G>A (m.902G>A) mutation in the ‘expectedly disruptive’ category.

Two more mutations map to this important structural element of the human mitoribosome. The extremely rare variation 232U > C (m.879T > C) was identified in a COX-deficient colonic crypt and in a cancer patient (33,50). The equivalent position in *E. coli*, *507C* is base paired to *524G* within a pseudoknot motif that provides structural stability to the 530 loop (Figs 2 and 3) (51). While the primary sequence of the pseudoknot is highly conserved, the identity of the bases constituting the *507:524* base pair is different in bacteria and human mitochondria. The bacterial pseudoknot was originally dissected by site-directed mutagenesis (51). It was shown that single base changes on either side of this structural element resulted in severe growth defects that could be restored by second-site, compensatory mutations (51). Interestingly, an A>G base change at the human equivalent of *G524*, namely 249A (m.896A) appears in 20 GenBank sequences and in a MITOMAP unpublished sequence (http://mitomap.org/bin/view.pl/MITOMAP/Submissions/20100804015). These observations suggest that certain mutations within the pseudoknot structure are tolerated in the organellar ribosome. A benign phenotype for 249A>G (m.896A>G) would not be in disagreement with HIA data and could be explained structurally. This is because G:U/U:G base-pair schemes within the pseudoknot are well tolerated in bacteria, including the very same scheme that would be induced by the 249A>G (m.896A>G) mutation (51,52).

The disruption of the bacterial pseudoknot, involving residues *507* and *524*, likely impairs the function of the *G530* in A-site decoding (47,48). In addition, disruption of the pseudoknot could also affect the maturation of SSU rRNA, as shown in *E. coli* (53). Taken together, these data allow the assignment of 232U>C (m.879T > C) within the ‘expectedly disruptive’ category, whereas 249A>G (m.896A>G) must be considered an ‘unlikely disruptive’ polymorphism.

### Helix 28

The 533U>G (m.1180T>G) mutation was identified in one pediatric subject with non-syndromic hearing loss (54). The *E. coli* equivalent is *G927* that is base paired to *U1390* in helix h28 (Figs 2 and 3). Next to this site is the universally conserved *G926* (conservation between 98 and 100%) (25), which is hydrogen bonded to the phosphate linking the P- and E-site codons on the mRNA (55) (Fig. 5E). In agreement with the important role of this region in translation, mutagenesis studies performed in yeast have shown that several base changes at the equivalent positions to *926*, *927* or *928* confer lethal phenotypes (56). Similarly, functional studies in bacteria demonstrated that all three mutations at *G926* drastically reduce the activity of the mutant ribosomes (57), albeit with only moderate effects on cell growth (58). Finally, b-h28 has been recently proposed to serve as the spring of the ratcheting process undergone by the ribosome during translocation (31). In summary, the available evidence obtained from different model organisms, points to an important involvement of h-28 residues in ribosomal function. As a result, the 533U>G (m.1180T > G) transversion is a good candidate for an ‘expectedly disruptive’ mutation.

### Helix 31

A C to G transversion has been identified at residue 579C (m.1226C) in a pediatric subject with non-syndromic hearing loss (54). Interestingly, a G>A transition at the adjacent 580G (m.1227G) was reported to be associated with schizophrenia in a single patient by Ueno *et al.* (59). While the 580G>A (m.1227G>A) mutation was properly placed on a phylogenetically derived, secondary-structure map of the mammalian mt-12S rRNA by the authors (59), no further inquiries regarding the structural and/or functional role of the residue were pursued. The *E. coli* equivalents to 579C (m.1226C) and 580G (m.1227G) are the universally conserved residue *C972* and the highly conserved *G973* (Figs 2 and 3). These residues map to the vicinity of the P- and A-site anticodon stem loops (ASLs) in the decoding site (Fig. 5F). Residue *C972* is base paired to *G963*, which is also universally conserved and places one of its phosphate oxygens <5 Å away from the backbone of the A-site ASL (Fig. 5F) (25,60). This base pair and the one formed by *G973* and *C962* appear to be important for the stabilization of the complicated structure of the loop atop helix b-h31 (Fig. 5F). Not surprisingly, mutations in this region have been found to disrupt ribosome activity in *E. coli*. For example, base changes at *C972*, *G963* and *A964* produced moderately deleterious, but dominant growth defects, whereas mutations at *G973* strongly impaired cell growth (61). Finally, the physical proximity of the *C972:G963* to the A-site ASL explains the observed effects of mutations in the region on the fidelity of A-site decoding (62). In summary, both the 579C > G (m.1226C > G) and the 580G>A (m.1227G>A) mutations are expected to be highly disruptive to the function of the mitoribosome.

### Helix 34

The mutations 620U>A (m.1267T>A) (63) and 676G>A (m.1323G>A) (64), mapping to mt-h34, have been identified in three cancer patients (see Supplementary Material, Table S1). This area of the ribosome has been implicated in the movement of the SSU head during translocation and in the binding of the translocation inhibitor spectinomycin (31,65,66). Indeed, the residue equivalent to 620U>A (m.1267T>A) in the chloroplasts of *Nicotiana tabacum* and of *Chlamydomonas* is a spectinomycin resistance (spec^R^) site (65). In *E. coli*, the equivalent residues to 620U>A (m.1267T > A) and 676G>A (m.1323G>A) are *1062U* and *1193G*, which base pair to *U1194* and *C1063*, respectively. Adjacent to the C*1063:G1193* base pair are residues *G1064* and *C1192* (Fig. 5G), which are part of the binding site for the antibiotic spectinomycin (55,66), an antibiotic proposed to restrict head movement by increasing the rigidity of the region around these residues (55,66). Despite their identity and location on the secondary structure map, *G1064* and *C1192* do not form a Watson–Crick base pair in the bacterial ribosome. Indeed, the human mt-SSU rRNA contains a U·C mismatch at the positions equivalent to *G1064* and *C1192* (compare Figs 2 and 3). Mutagenesis analysis of *G1064* and *C1192* has shown that mutations at both sites are well tolerated and confer resistance to spectinomycin (66,67). In contrast, mutations at *G1193*, also conferring spectinomycin resistance in *E. coli*, do so with a clear fitness cost (68). While no mutagenesis data exist for either *U1062* or its non-canonical base -pairing partner *U1194*, it is striking that the identity of both residues, forming a U·U mismatch, is conserved in human mitochondria. Analysis of the quaternary structure of the region shows that the N-terminal region of protein b-S3 makes direct contacts with the base of *U1062* and with the RNA backbone at *C1192* (Fig. 5G). The significance of this observation cannot be extrapolated to the organellar case, however, as a protein homologous to b-S3 is not present in the mitoribosome.

In summary, while some mutations in this region can be well tolerated in bacteria, others result in deleterious phenotypes. Specifically, the heterologous evidence appears quite solid for a potentially disruptive role of 676G>A (m.1323G>A). On the other hand, a prediction of the disruptive potential of 620U>A (m.1267T>A) cannot be based solely on the available heterologous data.

Another mt-h34 mutation, namely 680G>A (m.1327G>A), has been identified as homoplasmic in atrophic prostate epithelium (69). Its bacterial equivalent is position *1197*, which in *Thermus thermophilus* is also a G (A in *E. coli*). While mutagenesis studies at position *1197* have not been carried out, the following information provides evidence for an important role in translation. First, position *A1196* of *E. coli* has been cross linked to residues +5, +8 and +9 of the mRNA and shown to be protected by the binding of elongation factor G (70–72). Indeed, position *1196* is modeled in close proximity of the A-site codon–anticodon interaction in high-resolution structures of the bacterial ribosome (Fig. 5) (60). All these data taken together prompt us to consider 680G>A (m.1327G>A) as a ‘likely disruptive’ mutation.

### Helix 44

A U to C transition at position 852 (m.1499T>C) has been identified in a breast cancer patient (73). The equivalent position in *E. coli* would be *G1415* within b-h44 (Figs 2 and 3). *G1415* is base paired to *U1485*, which is part of inter-subunit bridge b-B3, a structure that appears to be conserved in the mitoribosome (Fig. 5H) (30,32). We have recently targeted the adjacent position *C1484* of *E. coli* SSU's rRNA for site-directed mutagenesis to find that alterations of the helical stability of this region can cause highly deleterious phenotypes (74). Similarly, mutations of the b-B3 partner region on the bacterial LSU also resulted in defective ribosomes (75), in agreement with the finding that bridge b-B3 is the pivot point for inter-subunit ratcheting (31). Thus, all the available evidence points at an important role of residue 852U (m.1499T) in the mitoribosome and argue for a likely disruptive role for the U to C transition at this position.

A highly interesting variation was recently listed in the MITOMAP unpublished variant database. The variation 910A>C (m.1557A>C) was identified in a Russian individual suffering from deafness (http://www.mitomap.org/bin/view.pl/MITOMAP/Submissions/20070709002). Its bacterial equivalent, the universally conserved *A1492* is, together with *A1493*, one of the most important residues of the ribosome. The functional role of these adenosines is to monitor the geometry of the codon–anticodon interaction at the A-site (Fig. 5H and I) (47,48). In bacteria, mutations at *A1492* or *A1493* result in dominant lethal phenotypes (57,76,77). Clearly, 910A>C (m.1557A>C) constitutes an extremely well-qualified candidate for a highly disruptive mutation.

The 915G>A (m.1562G>A) mutation was identified in a COX-deficient colonocyte in a study investigating clonally expanded mtDNA mutations associated with aging (78). The bacterial equivalent to this residue is the universally conserved *G1497*, adjacent to the P-site codon on the mRNA and is base paired to the also universally conserved *C1404* (Fig. 5H and I). The phosphate between *G1497* and *U1498* is within h-bonding distance of the 2′OH of position +1 of the mRNA, i.e. the first base of the P-site codon (60). Not surprisingly, mutagenesis studies showed that disrupting the *C1404:G1497* base pair resulted in serious impairment of tRNA binding at both P and A sites (79). Due to all these observations regarding the bacterial equivalent of 915G>A (m.1562G>A), the organellar mutation is clearly expected to result in a deleterious mitochondrial phenotype.

A 919C>U (m.1566C>T) transition has been identified in a cancer patient (41). The bacterial equivalent is the highly conserved *1501C*, which is base paired to *1401G* in the vicinity of the codon–anticodon interaction at the P site (Figs 2, 3 and 5I). Indeed, the adjacent *1400C* is stacked under position 34 of P-site tRNA (55). In agreement with their location, mutagenesis studies have shown that altering the *1401G:1501C* base pair completely abolishes ribosomal function (80,81). Hence, the heterologous evidence clearly places this mutation in the ‘expectedly disruptive’ category.

## DISCUSSION

Currently, all available structural information pertaining to the mammalian mitoribosome is contained within phylogenetically derived secondary structure maps of its rRNA components and in a single low-resolution reconstruction of the bovine organellar ribosome (25,82). Here, we have made the first systematic demonstration that this information can be combined with data obtained from heterologous sources to make predictions on the disruptive potential of mt-rRNA mutations. We believe that this exercise constitutes a valid first approximation toward understanding the role of these mutations in human disease. Indeed, we propose that these methods should become one of the prime functional tests for the examination of novel or rare mt-rRNA variants seen in the mtDNA of patients.

The predictive power of comparative analysis for the generation of structural predictions on the folding of large RNAs has been confirmed by the high-resolution structures of the ribosome (24,28). For this reason, it has been surprising to find that, except for one report (59), no attempt has been made to use phylogenetically derived secondary-structure maps for the interpretation of the available mutational data. This has, in some cases, resulted in quite inaccurate structural predictions that remain uncontested in the literature. For example, Mutai *et al*. (83) have used the program CENTROIDFOLD (84) to predict the disruptive potential of the 257C>U (m.904C>T) mutation. According to the authors, the C–U base change would disrupt the canonical G–C base pair between 242G and 257C (83). However, such a canonical base pair is not predicted by phylogenetic methods (Fig. 2) and is not substantiated by high-resolution data from heterologous sources, as shown in Supplementary Material, Figure S4.

### HIA and Ci analysis

In addition to HIA, we have also attempted to assign a Ci to all mutations, as described in the section Materials and Methods. This has been a common practice among researchers attempting to make sense of mutational data pertaining to mt-rRNAs. One of the main novelties of our Ci analysis is to include primary and secondary structure information from the region surrounding the tested residue (Ci_1ry_ and Ci_2ry_), as opposed to most reported Cis that merely reflect the conservation of said residue (54,83,85–87). Regarding conservation, we have measured this parameter across the width of phylogenetic tree (Ci_Univ_). In contrast, most authors have restricted their conservation analysis to a narrow range of phylogenetic space (54,83,85–87). While this practice works for other mitochondrial genes, we believe that the potential importance of the high number of exceptionally well-conserved residues of rRNA would be underestimated by Cis based on a narrow proportion of the total rRNA variation found on the tree of life. While our results show that Cis have some potential to predict the assignment of an important subset of mutations according to their HIA-predicted disruptive power, especially those within the ‘expectedly’ category, Cis cannot be relied upon for the assignment of pathogenicity to most mutations. One striking example is that of the ‘unlikely disruptive’ 249A>G (m.896A>G) mutation, for which all four Cis are well over the average value obtained for the ‘expectedly disruptive’ category. Similarly, some mutations within the ‘undetermined’ category display Ci scores that would place them within the ‘likely’ or even the ‘expectedly’ category, e.g. 67A>U(m.714A>T), 167A>G(m.814A>G) and 296G>A (m.943G>A). However, the best proof of the very limited predictive power of Cis in the context of mt-rRNA mutations is obtained when the ‘proven’ pathogenic mutations 847C>U (m.1494C>T) and 908A>G (m.1555A>G) are taken into account. The low Cis assigned to these mutations clearly demonstrate the limitations regarding their use as a way to obtain insightful information on the pathogenicity of mt-rRNA mutations.

In the case of universally conserved residues, both Ci_Univ_ and HIA predictions agree. It should be noted, however, that harmless or mild mutations at universally conserved rRNA residues do exist (58). As a result, the agreement between Ci_Univ_ and HIA predictions would be lost for such mutations. In summary, while the predictive potential of Cis remains an open issue, it appears clear that Ci analysis should always be applied in conjunction with HIA. This leads to a situation similar to that found while reporting mt-tRNA variants (6,7,10). Accordingly, a reliable assessment of pathogenicity of an mt-rRNA variant might require collaborative efforts between research centers to bring together the required analytical skills (9,88).

### Disruptive power and pathogenicity

How much damage to ribosomal function is necessary for pathogenicity, and how do other factors modulate the amount of damage caused by mt-rRNA variants? Here we make the assumption that ribosome disruption leads to defective organellar protein synthesis, which translates into mitochondrial dysfunction, and finally into clinical symptoms. This highly simplistic assumption, substantiated by an enormous amount of evidence both from the point of view of the ribosome and that of the mitochondrion, will serve as a first approximation toward the analysis of the pathogenic potential of mt-rRNA mutations. The only two mt-rRNA mutations whose pathogenicity has been established with certainty are 908A>G (m.1555A>G) and 847C>U (m.1494C>T). The bacterial equivalents of these residues, namely positions *1490* and *1410*, are part of the binding site for aminoglycoside antibiotics (Fig. 5H) (55). Despite their clear pathogenicity, when 908A>G (m.1555A>G) and 847C>U (m.1494C>T) mutations were recreated in bacterial systems, the cell growth was not completely arrested and the translation rate was not markedly decreased (89). Instead, a clear reduction in accuracy was observed in these studies (89). These results indicate that pathogenicity of mt-rRNA mutations can be achieved with ribosomes that can fully support translation, albeit with decreased accuracy. The fact that external modifiers, such as aminoglycoside antibiotics or nuclear markers, are needed to achieve the full pathogenic potential of 908A>G (m.1555A>G) and 847C>U (m.1494C>T) (21,90), suggests that, much like in the bacterial case, the mitochondrial mutations also result in functional ribosomes.

One would expect that mutations resulting in translation defects more severe than those conferred by 908A>G (m.1555A>G) and 847C>U (m.1494C>T) will not need the presence of additional factors to result in mitochondrial failure. Some examples of mutations expected to result in complete disruption of ribosomal function are contained within the ‘expectedly disruptive’ category. In principle, it seems reasonable to assume that ribosomes carrying severely disruptive mutations could co-exist in heteroplasmy with their wild-type counterparts. A similar co-existence of wild-type and mutant ribosomes has been frequently recreated in bacteria, where the dominance/recessiveness of highly deleterious mutations can be easily modulated by shifting the level of the mutant particles (91). The maintenance of bacterial growth is then dependent on the existence of enough wild-type ribosomes to support a sufficient level of protein synthesis. Extrapolating from the bacterial case, the heteroplasmy threshold above which the levels of disruptive mt-rRNA mutations would become incompatible with normal mitochondrial function would be expected to depend on two main factors. First, the severity of the deleterious effect caused by the base changes on the function of the mutated ribosomal particles (61,91,92). Secondly, the presence of phenotypic modifiers either *in cis* or *in trans* with the rRNA mutation. In the absence of direct biochemical data, a recent study in which the load of the 908A>G (m.1555A>G) mutation in a Chinese family was monitored becomes highly illustrative. The study showed that higher levels of heteroplasmy consistently resulted in more severe symptoms (93), thus mirroring the bacterial situation. In this study, heteroplasmy levels <20% were found in asymptomatic individuals, levels ∼50% in slightly affected individuals and >70% in severely affected patients (93).

Based on the large body of direct structural, mutational and functional data gathered in heterologous ribosomes, the mutations with the highest disruptive power described in this paper are 255G>A (m.902G>A) and 910A>C (m.1557A>C) (46) (http://www.mitomap.org/bin/view.pl/MITOMAP/Submissions/20070709002) which, in mitochondria, affect the key A-site residues in the mitochondria, equivalent to the bacterial *G530* and *1492A*, respectively (49,57,76). These nucleotides are amongst the most important ribosomal residues from the functional point of view and, therefore, it comes as no surprise that mutations at both *G530* and *1492A* have been found to cause dominant lethal phenotypes in bacteria (49,57,76). While in the case of the mitochondrial equivalent to *1492A*, namely 910A>C (m.1557A>C) (http://www.mitomap.org/bin/view.pl/MITOMAP/Submissions/20070709002), we lack data regarding heteroplasmy levels, the bacterial data strongly suggest that this mutation can only be present at low levels if the mitochondria are to retain their function (49,77). However, what constitutes the actual threshold for a defect caused by this mutation is currently unknown. As for the homoplasmic 255G>A (m.902G > A) (46), the fact that the all point mutations at its bacterial equivalent resulted in dominant lethal phenotypes, together with the central role of this residue in decoding, is a strong indication that all ribosomes in the mutant mitochondria are incapable of performing protein synthesis. As a result, this mutation meets all pathogenicity criteria proposed for a homoplasmic mutation: absence from healthy controls, remarkable phylogenetic conservation and unequivocal evidence of a functional defect of the mutant ribosome (9). While the evidence for a functional defect has been obtained in heterologous systems, the available data are so strong that pathogenicity can hardly be contested.

Two more ‘expectedly disruptive mutations’ 533U>G (m.1180T>G) and 579C>G (m.1226C>G) have also been found in homoplasmy (54). The disruption of the yeast equivalent to the mitochondrial U533:G827, formed between positions *927* and *1390* (bacterial numbering), gave rise to recessive lethal phenotypes. While such phenotypes are not as drastic as the dominant lethality caused by mutations at the bacterial G530 and A1492, the presence of 533U>G (m.1180T>G) in homoplasmy indicates that the affected mitochondria should be highly impaired in their ability to carry out protein synthesis, thus leading to mitochondrial dysfunction. The case of 579C>G (m.1226C>G) is different. Despite the fact that the Ci_Tot_ for this position is the same as that of 255G>A (m.902G>A) and higher than that of 910A>C (m.A1557A>C), it has been shown that disrupting the heterologous equivalent of the mitochondrial 570G:579C base pair, namely *b-963G:972C*, results in moderate dominant defects. Thus, the 579C>G (m.1226C>G) mutation is expected to result in at least suboptimal protein synthesis. Whether this can lead to enough organelle dysfunction to cause clinical phenotype remains unclear. The case of 579C>G (m.1226C>G) constitutes yet another example of the fact that Cis cannot be used to predict the disruptive potential of rRNA mutations even in the heterologous systems used as references in these studies. At the same time, this mutation underscores the enhanced discriminatory power gained by using HIA.

In summary, whilst a clear case for pathogenicity can be made for the 255G>A (m.902G>A) and the 533U>G (m.1180T>G) mutations, the 579C>G (m.1226C>G) mutation cannot be considered pathogenic by HIA alone.

### Role of HIA within a scoring system for mt-rRNA mutations

The existence of direct experimental data showing a mitochondrial defect caused by a mtDNA mutation is an essential pre-requisite of the current scoring systems to evaluate the pathogenicity of mt-tRNA and complex I gene mutations (6,7,9–11). In the absence of direct biochemical data, HIA provides an educated estimation of the disruptive potential of an important subset of mt-rRNA mutations. This situation is reminiscent of the pre-crystallography days (1990s) in which the study of ribosome structure had to rely on evidence obtained from low-resolution studies. Notably, the quality of HIA predictions can be substantially improved as additional heterologous data emerge. As such, this research provides new impetus for studies aimed at testing the effect of equivalent mutations in heterologous systems and constitutes an invitation to ‘ribosomologists’ to contribute to the improvement of HIA predictions, thus leading to higher diagnostic accuracy in mitochondrial clinics. An obvious first aim of such a collaborative effort between clinical and basic science should be to move as many of the ‘NEE’ mutations to the ‘likely disruptive’ or, perhaps, to the ‘expectedly disruptive’ category, by means of site-directed mutagenesis and biochemical studies in heterologous systems.

The importance of mutagenesis studies in heterologous systems as a way to ascertain the effects of mt-rRNA alteration studies is best exemplified by the case of the 908A>G (m.1555A>G) and 847C>U (m.1494C>T) mutations. When the mutations were introduced in bacteria, the observed phenotypes were in perfect agreement with the mitochondrial data (89,94). This, together with structural evidence (95), provided a molecular explanation to the pathological symptoms related to the mitochondrial mutations, namely that misreading of the genetic code in mitochondria can lead to a defective organelle (89). Therefore, HIA would clearly result in an ‘expectedly disruptive’ score for these mutations.

We have also shown that in cases where sufficient information exists regarding the genetic and pathological manifestation of the mutation, HIA alone can be used to predict the pathogenicity of certain mt-rRNA mutations. In these cases, the heterologous data are strong enough to replace direct biochemical evidence. In other cases, the disruptive potential predictions will be incorporated into a full-fledged scoring system for mt-rRNA that will allow an evaluation of their pathogenic potential. In addition to the usual elements, the mt-rRNA scoring system will include HIA predictions and CI scores. As mentioned above, the level of heteroplasmy, together with the degree of disruption of ribosomal function, will constitute major elements of the scoring system. Here again, future HIA-motivated studies performed in heterologous systems can provide extremely useful information. For example, quantitative studies with hybrid populations of ribosomes in bacteria (91) could be used to assess the heteroplasmy threshold at which mt-rRNA mutations become detrimental to normal protein synthesis.

### Final conclusions

Here, we have presented what we believe will be a central element of a scoring system for mt-rRNA mutations. HIA analysis appears to be a powerful tool that could help overcome the lack of biochemical and/or *trans*-mitochondrial cybrid data, which are considered to be crucial elements of the scoring systems for mt-tRNA and complex I genes (6,7,10,11). It should be noted that a simple ‘no-appearance-in-controls’ requisite, as established ever since the canonical criteria of DiMauro and Schon were established, will result in the discarding of well-known pathogenic mutations such as 908A>G (m.1555A>G) and 847C>U (m.1494C>T), underscoring the variability in penetrance of this group of mutations. One important challenge for the future must be to account for any effect of haplogroup background, as there is increasing evidence that this parameter is important in both the presentation and penetrance of mtDNA disease (22). Future analysis of mt-rRNA mutations will require that authors report their data fully including all the variants from the rCRS (particularly variants mapping to rRNA and tRNA genes), haplogroup definition, heteroplasmy levels, patient/control group sizes and data on the family/cohort structure. Similarly, mitochondrial genome sequences submitted to GenBank should clearly state whether the subject is considered a patient or a control.

While HIA predictions alone would not be sufficient for a definitive assignment of pathogenicity, except for some key residues with critical functional roles such as 255G>A (m.902G>A) and 533U>G (m.1180T>G), the method does provide a robust indicator of disruptiveness for a large group of mutations lacking direct evidence regarding their role in mitochondrial dysfunction. At the same time, HIA naturally allows the prioritization of variants for additional investigation.

## MATERIALS AND METHODS

### Nomenclature

Since the focus of this paper is the mitoribosome, all mt-rRNA base changes will be referred to by their gene, rather than their genomic position. For clarity, however, the genomic position will also be provided in brackets. Bacterial rRNA residues will be italicized throughout the text. Mitochondrial specific notation will be used to denote organellar base changes, e.g. m.1111A>G. Likewise, bacterial nomenclature will be used to denote bacterial base changes, i.e. *A1111G*. To distinguish structural elements in the bacterial and mitochondrial ribosomes their name will be preceded by b- and mt-, respectively. Small subunit helices are denoted by ‘h’ and large subunit helices are denoted by ‘H’.

### Sources of mutational data

The following sources of mutational data were used in this study: (a) scientific literature, (b) MITOMAP list of unpublished variants (1), (c) GenBank, (d) Phylotree (96) and (e) three unpublished variations (see below).

The criterion required for mt-rRNA mutations to be included in this study was their identification in human subjects, with either a suspected mitochondrial disease or somatic mtDNA mutations that had undergone clonal expansions in cancer patients, or COX-deficient cells located in aged tissues (97). In the case of studies involving tumorigenic tissue, the existence of non-cancerous control tissue from the same patient was a requirement. Similarly, the existence of COX-proficient surrounding tissue was a requirement in the case of COX-deficient samples of aged individuals. This allowed the variant to be identified as a somatic mutation that had undergone clonal expansion.

### mtDNA sequencing

Mutation 878C>G (m.1525C>G) was detected as follows. Total DNA was extracted from patient tissues using standard DNA extraction methodologies. The entire mitochondrial genome was amplified using 36 overlapping primer pairs as previously described (33). PCR products were cycle sequenced using ABI BigDye chemistry by following standard manufacturer's protocols and analyzed on an ABI3130xl genetic analyzer (Applied Biosystems). Mutations 125A>G (m.772A>G) and 361A>U (m.1008A>T) were identified as follows. The entire mtDNA of the patients was evaluated using a standard mitochip analysis (GeneChip*^®^* Human Mitochondrial Resequencing Array 2.0, Affymetrix, Inc.). Percentage heteroplasmy was quantified using the Pyrosequencing technology (Biotage AB, Uppsala, Sweden) on the PSQ96 MA platform according to the protocol of the manufacturer.

### Analysis of GenBank mitochondrial genome data

As of February 2013, there existed 17 850 human mtDNA sequences in GenBank [query = ‘Homo[Organism] + AND + gene_in_mitochondrion[PROP] + AND + 14 000:19000[SLEN] + NOT + pseudogene[All Fields]’ (98)]. All sequences were downloaded with Entrez Programming Utilities and our own PERL scripts (99). In addition, we also downloaded 210 sequences from http://www.phylotree.org/ that were not included in the GenBank collection as of May 2012. The data were downloaded in FASTA format and sequentially aligned to the Cambridge Reference (NC_012920.1) (100) by means of the MAFFT package (101), which provided a quick algorithm for massive sequence alignment. The generated alignments were then analyzed by means of a PERL script that recorded the total number of appearances for every variation. For variations with 15 or less appearances in the combined data set, including the data from both the GenBank and Phylotree databases, the possibility that the individual sequences could be linked to mitochondrial disease was investigated, by inspecting the related publication sources. In all cases, it was observed that no new patient sequences, other than the ones previously found in the literature, were present within GenBank.

### Assignment of the disruptive power potential by HIA

Phylogenetically derived, secondary-structure rRNA maps carry an enormous amount of conservation information that can be used to infer the relative positions of equivalent residues from organisms located at opposite ends of the universal phylogenetic tree. HIA uses this information as follows. First, comparison of phylogenetically derived secondary structures of the human mt-12S rRNA and the *E. coli* 16S rRNA (normally used as the standard numbering system for rRNA residues) (25) allowed the location of structurally equivalent residues. Secondly, once the position of a structurally equivalent residue was identified, its location in three dimensions (3D) was ascertained by visualization in high-resolution models of heterologous ribosomes (normally atomic models of the *E. coli* or *T. thermophilus* ribosomes). Subsequent analysis of the region surrounding the heterologous residue within the high-resolution structures provided a first approximation of the structural role of the mutated mitochondrial residue. Thirdly, an exhaustive search for additional structural and functional information regarding the heterologous residues was performed. Finally, the existence of a low-resolution reconstruction of the mitoribosome provided important clues regarding the conservation of gross features of the organellar particle.

The disruptive power potential was estimated for the equivalent heterologous positions by the following criteria: N = ‘certainly not disruptive’, supportive direct mutagenesis data in favor of this assignment exists for the tested residue or its base-pairing partner, U = ‘unlikely disruptive’, no direct mutagenesis data exist but enough indirect data exist in favor of this conclusion; NEE = ‘not enough evidence’, no direct or indirect evidence argues against a potential disruptive power; L = ‘likely disruptive’, no direct mutagenesis data exist but enough indirect data exist in favor of this conclusion and C = ‘certainly disruptive’, supportive direct mutagenesis data exist for the tested residue or its base -pairing partner. A mtDNA mutation would be considered to have equally disruptive power to its heterologous counterpart as long as the available structural data for the former is not in disagreement with the heterologous evidence, e.g. if the higher order structural elements taken into consideration are conserved in both the mitochondrial and heterologous ribosomes. Two additional categories were used to classify the mitochondrial mutations: und = ‘undetermined’, no heterologous data exist to evaluate the disruptive potential or the existing structural differences are too large to allow the extrapolation of conclusions made in the heterologous case and E = ‘expectedly disruptive’, which would contain the mitochondrial equivalents to residues assigned to the ‘certainly disruptive’ category in the heterologous system.

### Conservation index analysis

We have devised three simple conservation indexes (Cis). Ci_Univ_ was designed to monitor the universal conservation of the tested residue in SSU rRNAs encompassing the whole width of the universal phylogenetic tree, i.e. archaeal, bacterial, eukaryotic nuclear, mitochondria and chloroplast SSU rRNAs. It was calculated by assigning a value from 0 to 5 to the conservation categories defined by Cannone *et al.* (25) (see Table 2). Ci_1ry_ measures the degree of primary sequence conservation around the tested residue. It was calculated by assigning 1 point per matched residue within a five-residue window centered on the tested nucleotide. In addition, an extra point was assigned whenever the position of the heterologous residue had been predicted by primary sequence alignment between the human and *E. coli* SSU rRNA (102). Ci_2ry_ is similar to Ci_1ry_ but measures the degree of secondary structure around the tested residue. To calculate this index, we assumed structural equivalence between G:U/U:G and Watson–Crick base-pairing schemes, as assumed during the construction of phylogenetically derived secondary-structure maps (25). Again, 1 point was assigned per matched residue within the five-residue window, whenever the same secondary structure was predicted in phylogenetically derived secondary-structure maps of the human and *E. coli* SSU rRNA (25). A half-a-point penalty would be in place whenever a phylogenetically predicted mismatch was replaced by a canonical or G:U/U:G base pair, or vice versa. Finally, an arbitrary composite Ci was calculated as follows: Ci_Tot_ = (2 × Ci_Univ_) + (2 × Ci_1ry_) + Ci_2ry_. The weights of Ci_Univ_ and Ci_1ry_ were doubled to account for their higher predictive power (see Results).

### Structural analysis

The following crystal structures were obtained from rcsb (www.rcsb.org): 2I2P and 2I2T (39), 2J02 and 2J03 (60), 1VSA and 2OW8 (103), 2Y0U (104), 2XZM (27) and 3R8N (31). Structural analysis was performed with UCSF Chimera (105) and Phenix (106).

Figure 4 and Supplementary Material, Movie S1 were created as follows. The position of all mutations that had equivalents in *E. coli* 16S rRNA was located on the secondary-structure map of *T. thermophila* SSU (27). The *Tetrahymena* positions were then displayed on a 3D model of the SSU *T. thermophila* 18S rRNA that had been previously superposed onto the 3D model of the *E. coli* 16S rRNA with Chimera UCSF (27,106).

## SUPPLEMENTARY MATERIAL

Supplementary Material is available at *HMG* online.

## FUNDING

A.V.S. was supported by the Marie Curie Actions programme of the European Commission (Grant PIIF-GA-2010-274660) and the Spanish Ministerio de Economía y Competitividad (AGL2012-39274-C02-02). R.W.T., R.N.L. and Z.M.A.C.L. were supported by the Wellcome Trust (Strategic Award 096919/Z/11/Z). L.C.G. was supported by the Newcastle University Centre for Brain Ageing and Vitality supported by BBSRC, EPSRC, ESRC and MRC as part of the cross council Lifelong Health and Wellbeing Initiative (G0700718). J.L.E. was supported by an Academic fellowship from the Research Council UK. Funding to pay the Open Access publication charges for this article was provided by the Wellcome Trust (Strategic Award 096919/Z/11/Z).

## Supplementary Material

Supplementary Data
