# Supplementary material for: The role of the mitochondrial ribosome in human disease: searching for mutations in 12S mitochondrial rRNA with high disruptive potential
Source: Hum Mol Genet. 2013 Oct 2;23(4):949–67. doi: 10.1093/hmg/ddt490 (PMC3900107; doi:10.1093/hmg/ddt490)
Supplement: Supplementary Data [file supp_23_4_949__index.html]

The role of the mitochondrial ribosome in human disease: searching for mutations in 12S mitochondrial rRNA with high disruptive potential — The role of the mitochondrial ribosome in human disease: searching for mutations in 12S mitochondrial rRNA with high disruptive potential — Supplementary Data 

# The role of the mitochondrial ribosome in human disease: searching for mutations in 12S mitochondrial rRNA with high disruptive potential

## Supplementary Data

Supplementary Data

**Files in this Data Supplement:**

- Supplementary Data - Pdf file
- Supplementary Table 1 - pdf file
- Supplementary Table 2 - pdf file
- Supplementary Video - mp4 file
