# Supplementary material for: The role of the mitochondrial ribosome in human disease: searching for mutations in 12S mitochondrial rRNA with high disruptive potential
Source: Hum Mol Genet. 2013 Oct 2;23(4):949–67. doi: 10.1093/hmg/ddt490 (PMC3900107; doi:10.1093/hmg/ddt490)
Supplement: Supplementary Data [file supp_ddt490_ddt490supp.pdf]

Supporting Material for Manuscript entitled:

The role of the mitochondrial ribosome in human disease: Searching for mutations in 12S mitochondrial rRNA with high disruptive potential

by

Paul M. Smith[@], Joanna L. Elson[@], Laura C. Greaves, Saskia B. Wortmann, Richard J.T. Rodenburg, Robert N. Lightowlers, Zofia M. A. Chrzanowska-Lightowlers, Robert W. Taylor, Antón Vila-Sanjurjo

[@]Contributed equally to the work

SUPPLEMENTARY INFORMATION

Correlation between phylogenetic conservation and the distribution of variations in mt-12S rRNA:

In **Figure 1D** and **E** we showed that a clear correlation exists between the degree of conservation and the number of variations found in GenBank. A different way to plot the data in panels **D** and **E** of **Figure 1** is to calculate the number of variations which are present in the GenBank population above a particular threshold percentage. The results are shown below.

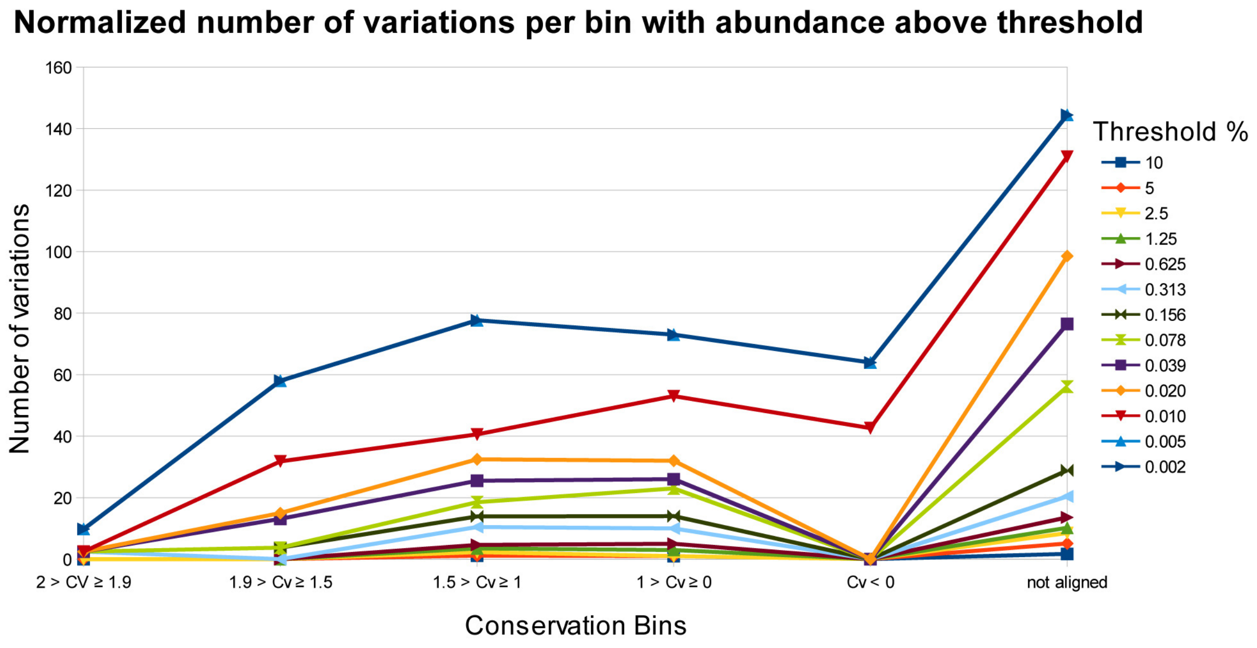

Supplementary Figure 1: Number of variations with abundance above different thresholds. The number of

per-**C_v**-bin variations above a particular abundance threshold was normalized as described in main text and plotted. Despite the discontinuity of the plotted data, a line graph representation was chosen for clarity.

Clearly, the less conserved bins, not only hold the vast majority of variations, as shown in **Figure 1D** and **E**, but also contain the variations with highest representation in the GenBank population (**Suppl. Figure 1**). Note that the **C_v<0** bin does not follow this trend for the reasons exposed in main text (*i.e.* too few data points). The plot demonstrates that the most abundant variations tend to accumulate in the least conserved rRNA bins, in agreement with our main hypothesis that that the distribution of variations in mt-12S rRNA is non-random and correlates with the phylogenetically derived conservation value, **C_v**.

As HIA controls, we have analyzed the only two alignable variations with an abundance ≥ 5% of the GenBank population, namely 791A>G (m.1438A>G) and 62G>A (m.709G>A). The 791A>G (m.1438A>G) variation, within the 1>**C_v**≥0 bin, appears in 16721 GenBank individuals. This is due to the use of the revised Cambridge Reference Sequence (rCRS), which does not carry the most abundant variant at this position. Accordingly, the actual variational abundance at this position should be 1129 G>A transitions, which amount to 6.33 % of the sequenced individuals in GenBank (The additional 193 sequences present at Phylotree were not used in our analysis (1)). Regarding the structure of mt-12S rRNA, the base change would replace a G:U wobble with a canonical A:U base pair, with minimal disruption of helical geometry (see **Figure 2**).

The 62G>A (m.709G>A) transition, with 2347 appearances is present in 13.2 % of the GenBank sequences. Its bacterial equivalent is *G107*. *G107* possesses a **C_v** of 1.148 and it is a G in 81.81% of all sequenced rRNAs (2). The next most abundant base at this position is A, with a percentage of 15.12 % (2). In the bacterial structure, N7 of *G107* is within hydrogen bonding distance of Lys 18 of protein *b-S20* (**Suppl. Figure 2A**), a protein that is missing in the organellar ribosome. Should an equivalent higher-order interaction exist with a yet unidentified residue that would involve the N7 of 62G in the human mitoribosome, need not necessarily be perturbed by the G>A replacement, perhaps explaining the conservation data. In addition, the N2 of *G107* has been modeled in close proximity of the N3 of *A60* (3.159Å in *Escherichia coli* and 2.811Å- 2.911Å in *Thermus thermophilus*) and the N1 of *A60* and the O2' of *G107* are within hydrogen bonding contact (2.601Å in *E. coli* and 2.740Å-3.010Å in *T. thermophilus*) (**Suppl. Figure 2A**, **B**). The presence of a tertiary interaction between the equivalents of the bacterial *A60* and *G107* appears to be important to stabilize the subunit's structure in the neighborhood of the almost universally conserved *A55*, which flips upon the binding of ternary complex during elongation (**Suppl. Figure 2B**) (3) . Since the equivalent to the bacterial *A60* in human mitochondria is residue 53A (m.700A), it is logical to think that a similar two-bonded tertiary interaction might exist between this residue and position 62G (m.709G) in mt-12S rRNA. Clearly, the 62G>A (m.709G>A) mutation would disrupt the base-to-base interaction with 53A.

In the two eukaryotic ribosomes for which we have high-resolution structures, *i.e. Saccharomyces cerevisiae* and *Tetrahymena thermophila* (RCSB accession numbers 3U5B and 2XZM), the equivalent to *b-A60* is a uridine (*U56* and *U55*, respectively) which places its O2 in close proximity of the guanine N2 at the position equivalent to the bacterial G107 (3.040Å to G91 and 2.505Å to G87, respectively; **Suppl. Figure 2C**) (4,5). In these two eukaryotic ribosomes the base-to-ribose hydrogen bond observed in the bacterial structures cannot be formed. These results indicate that a tertiary interaction between these residues formed by a single hydrogen bond is sufficient to maintain the structure of the region.

This observation suggests that the loss of one of the two hydrogen bonds presumably formed by 53A (m.700A) and 62G (m.709G), due to the G>A base change at the later residue, need not necessarily result in major structural defects.

In summary, the available heterologous evidence regarding the effect of the 62G>A (m.709G>A) and 791A>G (m.1438A>G) transitions supports the idea that the base changes can be easily accommodated without disruption of ribosomal function, in agreement with their observed high abundance.

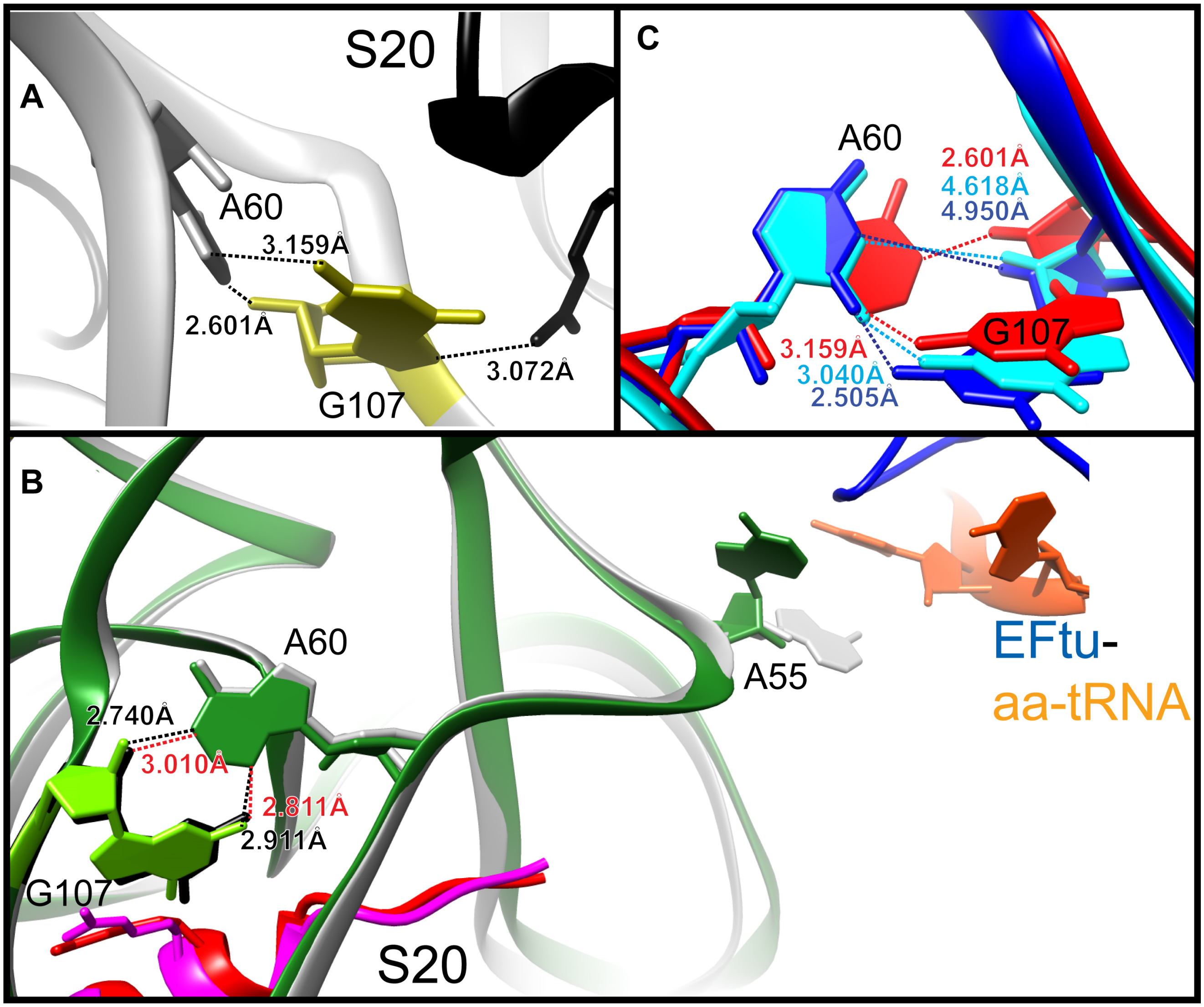

Supplementary Figure 2: Heterologous equivalents to 62G>A (m.709G>A). A. *E. coli*'s *G107* (yellow) and its interactions with *A60* and protein *b-S20* (black). RCSB accession code 2I2P. **B.** *T. thermophilus*'s *G107* in the factor-less and the EFtu-aa-tRNA-bound crystal structures of the 70S ribosome (RCSB accession code 2J00 and 2WRN, respectively). Residue *G107* (*E. coli* numbering) is shown in light green (2J00) and in black (2WRN) alongside with interatomic distances (2J00, black; 2WRN, red). Also shown are 16S rRNA (2J00, green; 2WRN, light grey), protein *b-S20* (2J00, magenta; 2WRN, red), and EFtu-aa-tRNA ternary complex (EFtu, blue; aa-tRNA,

orange). **C.** Interaction between the heterologous equivalents of 62G>A (m.709G>A) in eukaryotic ribosomes. Superposed structures of SSUs from *E. coli* (red, RCSB accession code 2I2P), *T. thermophila* (blue, RCSB accession code 2XZM), and *S. cerevisiae* (cyan, RCSB accession code 35UB). Inter-atomic distances are color coded.

<u>-HIA analysis of the 180A>G (m.827A>G) variation:</u>

The 180A>G (m.827A>G) base change occurs at a nearly invariant position (*A364* in *E. coli*, $\mathbf{C_v}$ = 1.935) and, for this reason, it was considered as an outlier during the analysis of the correlation between phylogenetic conservation and the distribution of variations in mt-12S rRNA. Despite its high degree of phylogenetic conservation, the 180A>G (m.827A>G) variation accounts for 1.85 % of the GenBank records. In the crystal structure of the *T. thermophilus* ribosome trapped in an intermediate state of translocation, the base of *A364* is within hydrogen bonding distance of the riboses of *C48*, through its N1 atom (2.976Å) and of *G362*, through its N7 atom (3.121Å) (**Suppl. Figure 3**, lower panel) (6). In *E. coli*, however, only *A364*'s N7 has been clearly modeled within direct contact distance of *G362* in some of the structures complexed with elongation factor EF-G (2.893Å) (**Suppl. Figure 3**, upper panel) (6,7). *A364* is part of a hairpin loop that directly interacts with conserved r-protein *b-S12* in the neighborhood of EF-G during translocation, an interaction proposed to be important for triggering GTP hydrolysis on the factor (6,7,8). Since no direct data regarding the role of *A364* is available, the importance of this highly conserved residue must be rationalized via the stabilization of local structure during translocation. Along these lines, it should be noted that at least the N7 interaction observed in *E. coli* would be compatible with an A to G base change. As a result, it is logical to think that the 180A>G (m.827A>G) variation need not necessarily have a deleterious effect on the local structure. While this would explain the high levels observed for the 180A>G (m.827A>G) variant in human mitochondria, the reason for the high phylogenetic conservation of this residue remains to be ascertained.

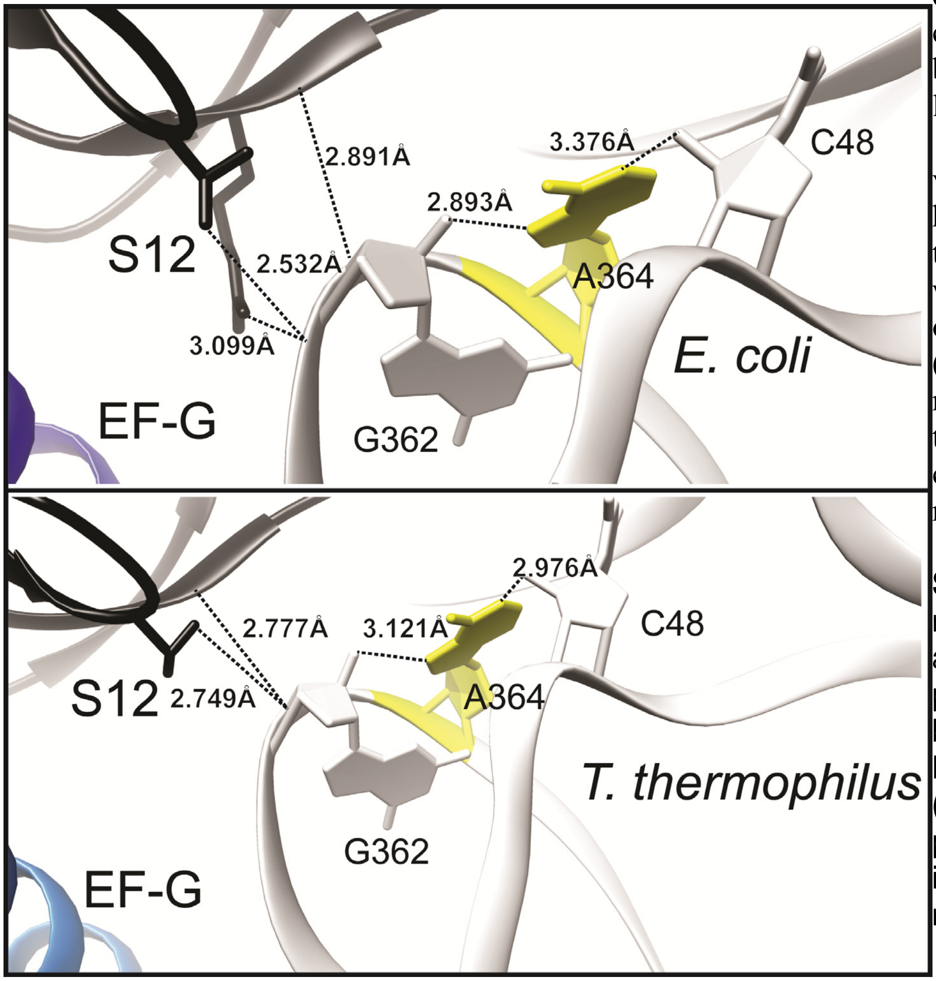

Supplementary Figure 3: *A364* region in *E. coli* (upper panel) and *T. thermophilus* (lower panel). Protein S12 shown in black. EF-G shown in blue. RCSB accession codes 4KJC (upper panel) and 4JUW (lower panel). Other residues mentioned in main text shown in gray. 16S rRNA shown in gray. Inter-atomic

distances are indicated (Note that ribbon representations do not necessarily reflect accurate inter-atomic distances involving backbone atoms).

ADDITIONAL SUPPLEMENTARY DATA

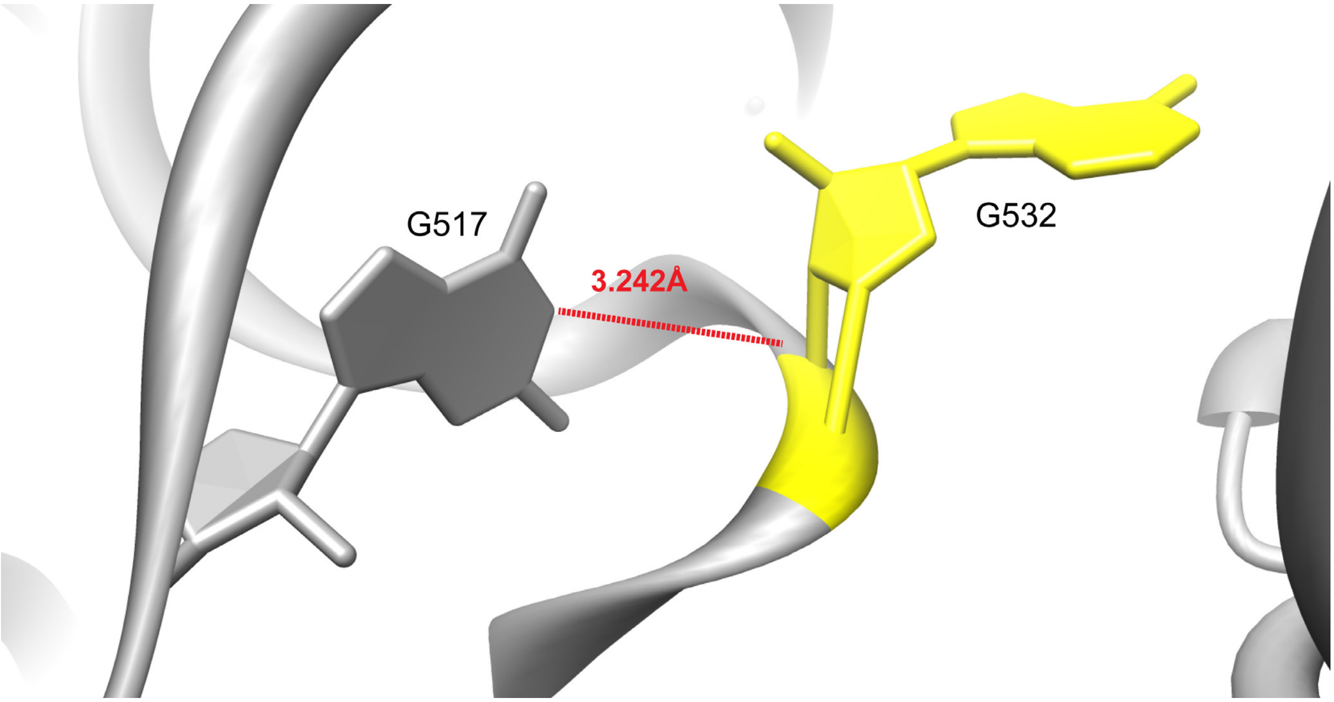

Supplementary Figure 4: Interaction between *G517* and *G532* of *T. thermophilus* 16S rRNA, equivalent to residues *242G* and *257C* in human mito-SSU-rRNA. Bacterial equivalents of mitochondrial mutations shown in yellow. Inter-atomic distances are indicated (Note that ribbon representations do not necessarily reflect accurate inter-atomic distances involving backbone atoms). RCSB accession code 2J00. Other residues mentioned in main text shown in gray. 16S rRNA shown in gray.

Supplementary Table I (provided separately)**:** Summary mutational data. The table summarizes the reported patient information regarding the mitochondrial 12S rRNA mutations analyzed in this work as well as the criteria used by the authors for the analysis of 12S rRNA structure. “nd” not determined. Referenced papers are shown parenthesized. Annotations for CI Criterion, taken literally from original sources, are as follows, 1 percentage of the human nucleotide variants with other 13 primates that have the wild-type nucleotide at that position, 2 conservation of nucleotide in human (H), bovine (B), mouse (M), and *Xenopus laevis* (X), (H/B/M/X), 3 blast search, and 4 based on the results of the multiple alignment of a wide range of mammalian sequences (51 sequences) by ClustalW.

Supplementary Movie 1 (provided separately)**:** Location of mitochondrial mutations in 3D structures of the *E. coli* and *T. thermophila* SSUs. E. coli 16S rRNA in red (accession code 2I2P); *T. thermophila* 18S rRNA in blue (accession code 2XZM). The predicted location for all *E. coli* nucleotides equivalent to mitochondrial mutations is shown as yellow sphere models, whereas the predicted location of the equivalent *T. thermophila* residues is shown as blue sphere models. In all cases, a perfect agreement between the structural predictions was found.

SUPPLEMENTARY REFERENCES:

1. van Oven M. and Kayser M. (2009) Updated comprehensive phylogenetic tree of global human mitochondrial DNA variation. *Hum. Mutat.,* 30, E386-94.

2. Cannone J.J., Subramanian S., Schnare M.N., Collett J.R., D'Souza L.M., Du Y., Feng B., Lin N., Madabusi L.V., Muller K.M. *et al.* (2002) The comparative RNA web (CRW) site: An online database of comparative sequence and structure information for ribosomal, intron, and other RNAs. *BMC Bioinformatics,* **3**, 2.

3. Schmeing T.M., Voorhees R.M., Kelley A.C., Gao Y.G., Murphy F.V.,4th, Weir J.R. and Ramakrishnan V. (2009) The crystal structure of the ribosome bound to EF-tu and aminoacyl-tRNA. *Science,* **326**, 688-694.

4. Rabl J., Leibundgut M., Ataide S.F., Haag A. and Ban N. (2011) Crystal structure of the eukaryotic 40S ribosomal subunit in complex with initiation factor 1. *Science,* **331**, 730-736.

5. Ben-Shem A., Garreau de Loubresse N., Melnikov S., Jenner L., Yusupova G. and Yusupov M. (2011) The structure of the eukaryotic ribosome at 3.0 A resolution. *Science,* **334**, 1524-1529.

6. Tourigny D.S., Fernandez I.S., Kelley A.C. and Ramakrishnan V. (2013) Elongation factor G bound to the ribosome in an intermediate state of translocation. *Science,* **340**, 1235490.

7. Pulk A. and Cate J.H. (2013) Control of ribosomal subunit rotation by elongation factor G. *Science,* **340**, 1235970.

8. Gao Y.G., Selmer M., Dunham C.M., Weixlbaumer A., Kelley A.C. and Ramakrishnan V. (2009) The structure of the ribosome with elongation factor G trapped in the posttranslocational state. *Science,* **326**, 694-699.
