# Supplementary material for: The role of the mitochondrial ribosome in human disease: searching for mutations in 12S mitochondrial rRNA with high disruptive potential
Source: Hum Mol Genet. 2013 Oct 2;23(4):949–67. doi: 10.1093/hmg/ddt490 (PMC3900107; doi:10.1093/hmg/ddt490)
Supplement: Supplementary Data [file supp_ddt490_ddt490supp_table1.pdf]

Source	Reported patient/ Cell line	mtDNA mutation	rRNA mutation	Reported associated clinical symptoms	Tissue	mtDNA mutation status	Haplotype	Reported CI criterion	Reported CI score	Reported secondary-structure analysis method	Reported predicted secondary structure
(1)	MiaPaCa2	**m.687G>A**	**40G>A**	pancreatic cancer	pancreatic cancer cell line	Homoplasmic	nd	nd	nd		nd
(2)	nd	**m.714A>T**	**67A>U**	Aminoglycoside-induced and nonsyndromic hearing loss	blood	Homoplasmic	nd	1	42.90%	RnaViz (108)	nd
(2)	nd	**m.737C>T**	**90C>U**	Aminoglycoside-induced and nonsyndromic hearing loss	blood	Homoplasmic	nd	1	71.40%	RnaViz (108)	nd
(3)	FZ117	**m.750G>C**	**103G>C**	Profound hearing impairment w/o aminoglycosides	blood	nd	B	2	A/G/G/-		nd
This work		**m.772A>G**	**125A>G**	myopathy	muscle	Homoplasmic	H2a2a1	nd	nd		nd
(4)	nd	**m.814A>G**	**167A>G**	obesity	blood	Homoplasmic	nd	nd	nd		nd
(5)	Patient 1 (crypt 7-)	**m.879T>C**	**232U>C**		COX-deficient colonic crypt	Heteroplasmic (50%)	nd	3	highly conserved		nd
(6)	PCA012	**m.879T>C**	**232U>C**	Prostate Cancer	benign and cancerous tissue	Heteroplasmic (60%)	T2	nd	nd		Non stem
(7)	nd	**m.896A>G**	**249A>G**		blood	nd	nd	nd	nd		nd
(8)		**m.902G>A**	**255G>A**		COX-deficient bladder patch	Homoplasmic	nd	nd	nd		nd
(9)	nd	**m.904C>T**	**257C>U**	hearing loss	blood	Homoplasmic	nd	4	48/51	Centroidfold (99)	Wt = C-G base-pair with G242. Mutant = disruptive wobble
(10)	Sample 1680	**m.912T>A**	**265U>A**	squamous cell cancers of the head and neck	tumor tissue and matched normal specimens	Heteroplasmic	nd	nd	nd		nd
(10)	Sample 1017	**m.924A>T**	**277A>U**	squamous cell cancers of the head and neck	tumor tissue and matched normal specimens	nd	nd	nd	nd		nd
(11)	nd	**m.929A>T**	**282A>U**	hearing loss	blood	Homoplasmic	nd	1	0.429	RnaViz (108)	↓A–U
(5)	Patient 3 (crypt 4-)	**m.943G>A**	**296G>A**		COX-deficient colonic crypt	Homoplasmic	nd	3	highly conserved		

(2)	nd	**m.955A>G**	**308A>G**	Aminoglycoside-induced and nonsyndromic hearing loss	blood	Homoplasmic	nd	1	0.643	RnaViz (108)	
(12)	CNSD96	**m.960C>A**	**313C>A**	Congenital Non-syndromic Deafness	blood	nd	nd	nd	nd		nd
(13)	A1	**m.961T>A**	**314U>A**	Deafness with Aminoglycoside Exposure	blood	nd	nd	nd	nd		nd
(14)	Patient ADPD29	**m.961InsC(5)**	**314InsC(5)**	Alzheimer's + Parkinson's disease		Homoplasmic	nd	nd	nd		nd
(2)	nd	**m.961delT**	**314delU**	Aminoglycoside-induced and nonsyndromic hearing loss	blood	Homoplasmic	nd	1	0.429	RnaViz (108)	
This work		**m.1008A>T**	**361A>U**	myopathy, behavioural problems	muscle	Homoplasmic	nd	nd	nd		nd
(15)	34804, 38494	**m.1011C>A**	**364C>A**	combined complex I + IV deficiency	Muscle	nd	nd	nd	nd		nd
(5)	Patient 1 (crypt 6-)	**m.1097G>A**	**450G>A**		COX-deficient colonic crypt	Homoplasmic	nd	blast search	moderatly conserved		
(16)		**m.1121A>G**	**474A>G**		COX-deficient colonocyte	Homoplasmic	nd	nd	nd		nd
(17)	Patient 14	**m.1132T>C**	**485U>C**	Breast cancer	cancerous and corresponding normal breast tissue	Heteroplasmic	J1c	nd	nd		nd
(18)	nd	**m.1154A>C**	**507A>C**	paternally inherited hearing loss	nd	Homoplasmic	nd	nd	nd		nd
(19)	Case 4	**m.1169G>A**	**522G>A**	Conventional renal carcinoma	primary renal carcinoma and matched control kidney cortex tissues	Heteroplasmic (>75%)	nd	nd	nd		nd
(20)	nd	**m.1180T>G**	**533U>G**	nonsyndromic hearing loss	blood	Homoplasmic	nd	2	T/T/T/T		nd
(20)	nd	**m.1226C>G**	**579C>G**	nonsyndromic hearing loss	blood	Homoplasmic	nd	2	C/C/C/C		nd
(21)	SP0066	**m.1227G>A**	**580G>A**	schizophrenia	blood	Heteroplasmic (60%)	F1	nd	nd	Comparative Methods	G:C to A●C
(22)	Patient B17	**m.1267T>A**	**620U>A**	Breast cancer	paired-tumor and adjacent normal tissue	Heteroplasmic	R30	nd	nd		nd
(5)	Patient 3 (crypt 1-)	**m.1289G>C**	**642G>C**		COX-deficient colonic crypt	Homoplasmic	nd	3	poorly conserved		nd
(10)	Sample 1680 ,	**m.1323G>A**	**676G>A**	Pancreatic cancer	primary head and neck tumor tissues and matched normal specimens (lymphocytes)	Heteroplasmic	nd	nd	nd		nd
(23)	case #3	**m.1323G>A**	**676G>A**	squamous cell cancer	Primary pancreatic tumour and matched normal tissue	Heteroplasmic	nd	nd	nd		nd

(24)		**m.1327G>A**	**680G>A**		COX-deficient atrophic prostate tissue	Homoplasmic	nd	nd	nd		nd
(25)	Sample HCT5	**m.1345G>A**	**698G>A**	Oncocytic lesions	50 oncocytic lesions and 52 control cases	Homoplasmic	nd	nd	nd		nd
(22)	Sample E13	**m.1345G>A**	**698G>A**	esophageal squamous cell carcinoma	paired-tumor and adjacent normal tissues from esophageal squamous cell carcinoma	Heteroplasmic	M2	nd	nd		nd
(26)	Sample C110	**m.1348G>A**	**701G>A**	esophageal cancer	cancerous and adjacent normal tissues	Heteroplasmic	FlaC	nd	nd		nd
(27)	Case 16	**m.1374A>T**	**727A>U**	dilated cardiomyopathy	blood	Homoplasmic	H33	nd	nd		nd
(28)	Patient 13	**m.1380G>A**	**733G>A**	chronic lymphatic leukaemia	blood with high white blood cell count & buccal epithelial cells as representative of normal tissue	Homoplasmic	nd	nd	nd		nd
(29)	Sample 1282	**m.1389G>A**	**742G>A**	Renal carcinoma	Fresh tumor and normal kidney parenchymal tissues	Heteroplasmic (50%)	nd	nd	nd		nd
(26)	Sample C095	**m.1389G>A**	**742G>A**	esophageal cancer	cancerous and adjacent normal tissues	Heteroplasmic	G2a	nd	nd		nd
(30)	Patient 797	**m.1499T>C**	**852U>C**	Breast cancer	Breast cancer samples and corresponding nontumorous breast tissue	Heteroplasmic (50%)	nd	nd	nd		nd
This work	nd	**m.1525C>G**	**878C>G**	Profound hearing loss	nd	nd	nd	nd	nd		nd
(2)	nd	**m.1535T>C**	**888U>C**	hearing loss	blood	Homoplasmic	nd	1	42.90%	RnaViz (108)	↓U–A
(31)	Case E05	**m.1544A>T**	**897A>U**	esophageal cancer	Paired tumors and surrounding tissues	Heteroplasmic	nd	nd	nd		Disruption of a critical basepair in a stem region
(32)	Sample S25	**m.1557A>C**	**910A>C**	Deafness	blood	nd	nd	nd	nd		nd
(16)		**m.1562G>A**	**915G>A**		COX-deficient colonocyte	Heteroplasmic (50%)	nd	nd	nd		nd
(19)	Case 6	**m.1566C>T**	**919C>U**	Papillary Renal Carcinoma	Tumour and the corresponding healthy cortex tissue	Heteroplasmic (<25%)	nd	nd	nd		nd
(16)		**m.1576G>A**	**929G>A**		COX-deficient colonocyte	Heteroplasmic (50%)	nd	nd	nd		nd
(5)	Patient 4 (crypt 7-)	**m.1586G>A**	**939G>A**		COX-deficient colonic crypt	Heteroplasmic (60%)	nd	3	highly conserved		nd

References

1. Jones J.B., Song J.J., Hempen P.M., Parmigiani G., Hruban R.H. and Kern S.E. (2001) Detection of mitochondrial DNA mutations in pancreatic cancer offers a "mass"-ive advantage over detection of nuclear DNA mutations. Cancer Res., 61, 1299-1304.
2. Lu J., Li Z., Zhu Y., Yang A., Li R., Zheng J., Cai Q., Peng G., Zheng W., Tang X. et al. (2010) Mitochondrial 12S rRNA variants in 1642 han chinese pediatric subjects with aminoglycoside-induced and nonsyndromic hearing loss. Mitochondrion, 10, 380-390.
3. Zhu Y., Li Q., Chen Z., Kun Y., Liu L., Liu X., Yuan H., Zhai S., Han D. and Dai P. (2009) Mitochondrial haplotype and phenotype of 13 chinese families may suggest multi-original evolution of mitochondrial C1494T mutation. Mitochondrion, 9, 418-428.
4. Guo L.J., Oshida Y., Fuku N., Takeyasu T., Fujita Y., Kurata M., Sato Y., Ito M. and Tanaka M. (2005) Mitochondrial genome polymorphisms associated with type-2 diabetes or obesity. Mitochondrion, 5, 15-33.
5. Taylor R.W., Barron M.J., Borthwick G.M., Gospel A., Chinnery P.F., Samuels D.C., Taylor G.A., Plusa S.M., Needham S.J., Greaves L.C. et al. (2003) Mitochondrial DNA mutations in human colonic crypt stem cells. J. Clin. Invest., 112, 1351-1360.
6. Kloss-Brandstatter A., Schafer G., Erhart G., Huttenhofer A., Coassin S., Seifarth C., Summerer M., Bektic J., Klocker H. and Kronenberg F. (2010) Somatic mutations throughout the entire mitochondrial genome are associated with elevated PSA levels in prostate cancer patients. Am. J. Hum. Genet., 87, 802-812.
7. Voets A. and Smeets H. (2010) MITOMAP mtDNA Sequence Data: Unpublished Variant 20100804015. (http://www.mitomap.org/bin/view.pl/MITOMAP/Submissions/20100804015)
8. Gaisa N.T., Graham T.A., McDonald S.A., Canadillas-Lopez S., Poulsom R., Heidenreich A., Jakse G., Tadrous P.J., Knuechel R. and Wright N.A. (2011) The human urothelium consists of multiple clonal units, each maintained by a stem cell. J. Pathol., 225 , 163-171.
9. Mutai H., Kouike H., Teruya E., Takahashi-Kodomari I., Kakishima H., Taiji H., Usami S., Okuyama T. and Matsunaga T. (2011) Systematic analysis of mitochondrial genes associated with hearing loss in the japanese population: DHPLC reveals a new candidate mutation. BMC Med. Genet., 12, 135.
10. Zhou S., Kachhap S., Sun W., Wu G., Chuang A., Poeta L., Grumbine L., Mithani S.K., Chatterjee A., Koch W. et al. (2007) Frequency and phenotypic implications of mitochondrial DNA mutations in human squamous cell cancers of the head and neck. Proc. Natl. Acad. Sci. U. S. A., 104, 7540-7545.
11. Shen Z., Zheng J., Chen B., Peng G., Zhang T., Gong S., Zhu Y., Zhang C., Li R., Yang L. et al. (2011) Frequency and spectrum of mitochondrial 12S rRNA variants in 440 han chinese hearing impaired pediatric subjects from two otology clinics. J. Transl. Med., 9, 4.
12. Lingala H.B. and Thangaraj K. (2011) MITOMAP mtDNA Sequence Data: Unpublished Variant 20110503002. (http://www.mitomap.org/bin/view.pl/MITOMAP/Submissions/20110503002)
13. Dzhemileva L.U. and Khusnutdinova E.K. (2003) MITOMAP mtDNA Sequence Data: Unpublished Variant 20060913001. (http://www.mitomap.org/bin/view.pl/MITOMAP/Submissions/20060913001)
14. Shoffner J.M., Brown M.D., Torroni A., Lott M.T., Cabell M.F., Mirra S.S., Beal M.F., Yang C.C., Gearing M. and Salvo R. (1993) Mitochondrial DNA variants observed in alzheimer disease and parkinson disease patients. Genomics, 17 , 171-184.
15. Freitag M., Holger P. and Meitinger T. (2011) MITOMAP mtDNA Sequence Data: Unpublished Variant 20110302015. (http://www.mitomap.org/bin/view.pl/MITOMAP/Submissions/20110302015)
16. Greaves L.C., Elson J.L., Nooteboom M., Grady J.P., Taylor G.A., Taylor R.W., Mathers J.C., Kirkwood T.B. and Turnbull D.M. (2012) Comparison of mitochondrial mutation spectra in ageing human colonic epithelium and disease: Absence of evidence for purifying selection in somatic

mitochondrial DNA point mutations. PLoS Genet., 8 , e1003082.
17. Fendt L., Niederstatter H., Huber G., Zelger B., Dunser M., Seifarth C., Rock A., Schafer G., Klocker H. and Parson W. (2011) Accumulation of mutations over the entire mitochondrial genome of breast cancer cells obtained by tissue microdissection. Breast Cancer Res. Treat., 128, 327-336.
18. Konings A., Van Camp G., Goethals A., Van Eyken E., Vandevelde A., Ben Azza J., Peeters N., Wuyts W., Smeets H. and Van Laer L. (2008) Mutation analysis of mitochondrial DNA 12SrRNA and tRNASer(UCN) genes in non-syndromic hearing loss patients. Mitochondrion, 8, 377-382.
19. Meierhofer D., Mayr J.A., Fink K., Schmeller N., Kofler B. and Sperl W. (2006) Mitochondrial DNA mutations in renal cell carcinomas revealed no general impact on energy metabolism. Br. J. Cancer, 94, 268-274.
20. Li R., Greinwald J.H.,Jr, Yang L., Choo D.I., Wenstrup R.J. and Guan M.X. (2004) Molecular analysis of the mitochondrial 12S rRNA and tRNASer(UCN) genes in paediatric subjects with non-syndromic hearing loss. J. Med. Genet., 41, 615-620.
21. Ueno H., Nishigaki Y., Kong Q.P., Fuku N., Kojima S., Iwata N., Ozaki N. and Tanaka M. (2009) Analysis of mitochondrial DNA variants in japanese patients with schizophrenia. Mitochondrion, 9, 385-393.
22. Gochhait S., Bhatt A., Sharma S., Singh Y.P., Gupta P. and Bamezai R.N. (2008) Concomitant presence of mutations in mitochondrial genome and p53 in cancer development - a study in north indian sporadic breast and esophageal cancer patients. Int. J. Cancer, 123, 2580-2586.
23. Kassauei K., Habbe N., Mullendore M.E., Karikari C.A., Maitra A. and Feldmann G. (2006) Mitochondrial DNA mutations in pancreatic cancer. Int. J. Gastrointest. Cancer., 37, 57-64.
24. Gaisa N.T., Graham T.A., McDonald S.A., Poulsom R., Heidenreich A., Jakse G., Knuechel R. and Wright N.A. (2011) Clonal architecture of human prostatic epithelium in benign and malignant conditions. J. Pathol., 225, 172-180.
25. Gasparre G., Porcelli A.M., Bonora E., Pennisi L.F., Toller M., Iommarini L., Ghelli A., Moretti M., Betts C.M., Martinelli G.N. et al. (2007) Disruptive mitochondrial DNA mutations in complex I subunits are markers of oncocytic phenotype in thyroid tumors. Proc. Natl. Acad. Sci. U. S. A., 104, 9001-9006.
26. Liu J., Wang L.D., Sun Y.B., Li E.M., Xu L.Y., Zhang Y.P., Yao Y.G. and Kong Q.P. (2012) Deciphering the signature of selective constraints on cancerous mitochondrial genome. Mol. Biol. Evol., 29, 1255-1261.
27. Zaragoza M.V., Fass J., Diegoli M., Lin D. and Arbustini E. (2010) Mitochondrial DNA variant discovery and evaluation in human cardiomyopathies through next-generation sequencing. PLoS One, 5, e12295.
28. He L., Luo L., Proctor S.J., Middleton P.G., Blakely E.L., Taylor R.W. and Turnbull D.M. (2003) Somatic mitochondrial DNA mutations in adult-onset leukaemia. Leukemia, 17, 2487-2491.
29. Nagy A., Wilhelm M., Sukosd F., Ljungberg B. and Kovacs G. (2002) Somatic mitochondrial DNA mutations in human chromophobe renal cell carcinomas. Genes Chromosomes Cancer, 35, 256-260.
30. Tseng L.M., Yin P.H., Yang C.W., Tsai Y.F., Hsu C.Y., Chi C.W. and Lee H.C. (2011) Somatic mutations of the mitochondrial genome in human breast cancers. Genes Chromosomes Cancer, 50, 800-811.
31. Tan D.J., Chang J., Liu L.L., Bai R.K., Wang Y.F., Yeh K.T. and Wong L.J. (2006) Significance of somatic mutations and content alteration of mitochondrial DNA in esophageal cancer. BMC Cancer, 6, 93.
32. Tazetdinov A.M., Dzhemileva L.U., Ponidelko S.N., Markova T.G. and Khusnutdinova E.K. (2007) MITOMAP mtDNA Sequence Data: Unpublished Variant 20070709002. (http://www.mitomap.org/bin/view.pl/MITOMAP/Submissions/20070709002)
