# Supplementary material for: The role of the mitochondrial ribosome in human disease: searching for mutations in 12S mitochondrial rRNA with high disruptive potential
Source: Hum Mol Genet. 2013 Oct 2;23(4):949–67. doi: 10.1093/hmg/ddt490 (PMC3900107; doi:10.1093/hmg/ddt490)
Supplement: Supplementary Data [file supp_ddt490_ddt490supp_table2.pdf]

E. coli 16S rRNA (J01695)	conservation value for that position (C_v)*	Mitochondrial genomic position	mt-12S rRNA position	sequence	No. appearances in GenBank
1 A	-0.854				
2 A	-0.9				
3 A	-0.718				
4 U	-0.885				
5 U	-0.686				
6 G	-0.392	648	1	A	2
7 A	0.49	649	2	A	
8 A	0.666	650	3	T	16
9 G	0.52	651	4	A	
10 A	0.437	652	5	G	
11 G	1.609	653	6	G	1
12 U	0.79	654	7	T	4
13 U	1.802	655	8	T	
14 U	1.771	656	9	T	3
15 G	1.686	657	10	G	
16 A	1.347	658	11	G	1
17 U	1.919	659	12	T	
18 C	1.621	660	13	C	
19 A	1.38	661	14	C	
20 U	1.777	662	15	T	
21 G	1.498	663	16	A	529
22 G	0.88	664	17	G	
23 C	1.842	665	18	C	
24 U	0.561	666	19	C	
25 C	0.643	667	20	T	
26 A	0.988	668	21	T	
27 G	0.832	669	22	T	43
28 A	0.506	670	23	C	
29 U	0.386	671	24	T	
30 U	0.141	672	25	A	
31 G	0.024				
32 A	1.004	673	26	T	
33 A	1.166	674	27	T	
34 C	0.741	675	28	A	
35 G	1.57	676	29	G	11
36 C	1.45	677	30	C	
37 U	1.849	678	31	T	7
38 G	0.536	679	32	C	2
39 G	1.171	680	33	T	57

40 C	0.927	681	34	T	27
41 G	0.741	682	35	A	
42 G	0.772	683	36	G	
43 C	0.935	684	37	T	1
44 A	0.728	685	38	A	
45 G	0.286	686	39	A	2
46 G	1.047	687	40	G	
47 C	0.749	688	41	A	
48 C	0.702	689	42	T	2
49 U	1.886	690	43	T	
50 A	1.336				
51 A	1.172	691	44	A	
52 C	0.534	692	45	C	
53 A	1.07	693	46	A	
54 C	1.917	694	47	C	
55 A	1.942	695	48	A	
56 U	1.96	696	49	T	
57 G	1.905	697	50	G	
58 C	1.868	698	51	C	
59 A	1.665	699	52	A	2
60 A	1.419	700	53	A	
61 G	1.791	701	54	G	
62 U	1.642	702	55	C	3
	-	703	56	A	
	-	704	57	T	
	-	705	58	C	
	-	706	59	C	
63 C	0.79				
64 G	0.381				
65 A	0.554				
66 A	0.278				
67 C	0.409				
68 G	0.521				
69 G	-0.552				
70 U	-0.919				
71 A	-0.565				
72 A	-0.614				
73 C	-0.728				
74 A	-0.699				
75 G	-0.764				
76 G	-0.803				
77 A	-0.894				
78 A	-0.962				

79 G	-0.947				
80 A	-0.872				
81 A	-0.824				
82 G	-0.684				
83 C	-0.516				
84 U	0.272				
85 U	-0.339				
86 G	-0.242				
87 C	-0.593				
88 U	-0.767				
89 U	-0.896				
90 C	-0.964				
91 U	-0.961				
92 U	-0.894				
93 U	-0.793				
94 G	-0.501				
95 C	-0.83				
96 U	-0.815				
97 G	-0.71				
98 A	-0.847				
99 C	-0.668				
100 G	-0.623				
101 A	0.901				
102 G	0.469				
103 U	0.337				
104 G	0.504				
105 G	1.664	707	60	C	
106 C	1.751	708	61	C	1
107 G	1.148	709	62	G	2347
108 G	0.32	710	63	T	196
109 A	1.414	711	64	T	44
110 C	1.201	712	65	C	1
111 G	1.19	713	66	C	
112 G	1.406	714	67	A	4
113 G	1.26	715	68	G	1
114 U	1.855	716	69	T	
115 G	1.224	717	70	G	
116 A	1.328	718	71	A	
117 G	1.098	719	72	G	326
118 U	1.383	720	73	T	3
	-	721	74	T	45
	-	722	75	C	13
	-	723	76	A	69

-	724	77	C	
-	725	78	C	
-	726	79	C	
-	727	80	T	
-	728	81	C	1
-	729	82	T	1
-	730	83	A	
-	731	84	A	19
-	732	85	A	16
-	733	86	T	
-	734	87	C	
-	735	88	A	19
-	736	89	C	5
-	737	90	C	
-	738	91	A	3
-	739	92	C	15
-	740	93	G	22
-	741	94	A	
-	742	95	T	10
-	743	96	C	1
-	744	97	A	1
-	745	98	A	26
-	746	99	A	
-	747	100	A	3
-	748	101	G	3
-	749	102	G	3
-	750	103	A	17575
-	751	104	A	7
-	752	105	C	72
-	753	106	A	

119 A	**1.835**
120 A	**0.921**
121 U	**0.524**
122 G	**0.55**
123 U	**1.508**
124 C	**0.418**
125 U	**0.837**
126 G	**0.168**
127 G	**0.378**
128 G	**0.528**
129 A	**0.26**
130 A	**1.039**
131 A	**0.507**
132 C	**0.53**
133 U	**1.043**

134 G	**0.068**
135 C	**0.501**
136 C	**0.686**
137 U	**0.107**
138 G	**-0.311**
139 A	**-0.346**
140 U	**-0.348**
141 G	**-0.073**
142 G	**0.185**
143 A	**0.175**
144 G	**-0.367**
145 G	**-0.332**
146 G	**0.376**
147 G	**1.148**
148 G	**0.533**
149 A	**1.175**
150 U	**0.727**
151 A	**1.222**
152 A	**0.528**
153 C	**0.685**
154 U	**-0.326**
155 A	**-0.474**
156 C	**-0.311**
157 U	**-0.222**
158 G	**0.268**
159 G	**0.598**
160 A	**1.273**
161 A	**0.74**
162 A	**0.662**
163 C	**0.293**
164 G	**-0.255**
165 G	**-0.289**
166 U	**-0.277**
167 A	**-0.332**
168 G	**0.323**
169 C	**0.414**
170 U	**1.137**
171 A	**1.214**
172 A	**1.221**
173 U	**1.002**
174 A	**0.82**
175 C	**1.134**
176 C	**0.253**
177 G	**0.134**
178 C	**-0.22**

179 A	**0.486**
180 U	**0.593**
181 A	**0.173**
182 A	**-0.787**
183 C	**-0.668**
184 G	**-0.806**
185 U	**-0.601**
186 C	**-0.246**
187 G	**-0.396**
188 C	**-0.691**
189 A	**-0.395**
190 A	**-0.545**
191 G	**-0.062**
192 A	**-0.549**
193 C	**-0.73**
194 C	**-0.438**
195 A	**0.601**
196 A	**0.626**
197 A	**0.61**
198 G	**0.326**
199 A	**-0.788**
200 G	**-0.937**
201 G	**-0.602**
202 G	**-0.591**
203 G	**-0.705**
204 G	**-0.471**
205 A	**-0.467**
206 C	**-0.577**
207 C	**-0.547**
208 U	**-0.306**
209 U	**-0.253**
210 C	**-0.536**
211 G	**-0.603**
212 G	**-0.528**
213 G	**-0.58**
214 C	**-0.66**
215 C	**-0.605**
216 U	**-0.701**
217 C	**-0.938**
218 U	**-0.73**
219 U	**0.058**
220 G	**0.218**
221 C	**0.045**

222 C	-0.165				
223 A	-0.428				
224 U	-0.343				
225 C	-0.507				
226 G	0.306				
227 G	1.041				
228 A	0.622				
229 U	0.209				
230 G	0.529				
231 U	0.5				
232 G	0.58				
233 C	0.523				
234 C	0.367				
235 C	0.335				
236 A	0.647				
237 G	0.438				
238 A	1.534				
239 U	0.207				
240 G	0.58	754	107	A	
241 G	0.173	755	108	G	2
242 G	0.425	756	109	C	
243 A	1.847	757	110	A	
244 U	1.882	758	111	T	
245 U	0.926	759	112	C	
246 A	1.899	760	113	A	1
247 G	1.319	761	114	A	2
248 C	0.873	762	115	G	
249 U	1.439	763	116	C	
250 A	0.787	764	117	A	
251 G	1.454	765	118	C	
252 U	1.023	766	119	G	
253 A	1.076	767	120	C	
254 G	1.219	768	121	A	
	-	769	122	G	1671
	-	770	123	C	10
	-	771	124	A	1
	-	772	125	A	
255 G	1.158				
256 U	0.85				
257 G	0.409				
258 G	0.303				
259 G	0.938				
260 G	0.984				

261 U	1.368				
262 A	1.278				
263 A	0.894				
264 C	-0.095				
265 G	0.804				
266 G	1.053				
267 C	0.909				
268 U	0.269				
269 C	0.273				
270 A	0.995				
271 C	1.041				
272 C	1.079	773	126	T	1
273 U	1.065	774	127	G	
274 A	0.997	775	128	C	
	-	776	129	A	
275 G	1.815	777	130	G	
276 G	0.891	778	131	C	
277 C	1.317	779	132	T	1
278 G	0.279	780	133	C	
279 A	0.961	781	134	A	
280 C	0.679	782	135	A	
281 G	1.182	783	136	A	3
282 A	1.913	784	137	A	
283 U	0.942	785	138	C	
284 C	0.565	786	139	G	
285 C	0.172	787	140	C	
286 C	0.588	788	141	T	
287 U	1.845	789	142	T	28
288 A	1.471	790	143	A	
289 G	0.965	791	144	G	
290 C	1.042	792	145	C	1
	-	793	146	C	10
	-	794	147	T	13
	-	795	148	A	2
291 U	-0.19				
292 G	0.809				
293 G	0.268				
294 U	-0.163				
295 C	0.199				
296 U	1.204				
297 G	0.535				
298 A	1.157				
299 G	1.308				

300 A	1.077				
301 G	1.247				
302 G	0.335				
303 A	-0.113				
304 U	0.116				
305 G	1.089				
306 A	0.768				
307 C	-0.003				
308 C	0.719				
309 A	-0.114				
310 G	1.007	796	149	G	1
311 C	0.95	797	150	C	
312 C	1.213	798	151	C	
313 A	1.863	799	152	A	
314 C	1.275	800	153	C	
315 A	1.801	801	154	A	5
316 C	-0.066				
317 U	0.489				
318 G	1.187				
319 G	0.605				
320 A	0.184				
321 A	0.542				
322 C	1.103				
323 U	1.221				
324 G	1.179				
325 A	1.145				
326 G	1.074				
327 A	1.212				
328 C	0.126				
329 A	1.172				
330 C	0.882				
331 G	1.013				
332 G	1.056				
333 U	0.165				
334 C	0.591				
335 C	0.609				
336 A	0.413				
337 G	0.05				
338 A	0.877				
339 C	1.641	802	155	C	
340 U	0.769	803	156	C	
341 C	1.206	804	157	C	1
342 C	1.593	805	158	C	

343 U	1.109	806	159	C	1
344 A	1.804	807	160	A	
345 C	1.195	808	161	C	2
346 G	1.784	809	162	G	
347 G	1.82	810	163	G	
348 G	1.246	811	164	G	1
349 A	1.349	812	165	A	
350 G	1.188	813	166	A	100
351 G	1.289	814	167	A	1
352 C	1.909	815	168	C	
353 A	1.831	816	169	A	
354 G	1.881	817	170	G	
355 C	1.94	818	171	C	
356 A	1.966	819	172	A	
357 G	1.935	820	173	G	
358 U	1.147	821	174	T	
359 G	0.515	822	175	G	
360 G	1.231	823	176	A	
361 G	0.662	824	177	T	13
362 G	1.356	825	178	T	1
363 A	1.229	826	179	A	
364 A	1.935	827	180	A	330
365 U	0.977	828	181	C	
366 A	0.785	829	182	C	1
367 U	1.724	830	183	T	
368 U	1.959	831	184	T	
369 G	0.407	832	185	T	
370 C	0.578	833	186	A	
371 A	0.324	834	187	G	1
372 C	1.775	835	188	C	
373 A	1.486	836	189	A	
374 A	1.75	837	190	A	
375 U	1.937	838	191	T	
376 G	0.97	839	192	A	2
377 G	0.795	840	193	A	
378 G	0.605	841	194	A	
379 C	0.672	842	195	C	
380 G	0.97	843	196	G	
381 C	0.603	844	197	A	
382 A	1.204	845	198	A	
383 A	1.616	846	199	A	
384 G	0.627	847	200	G	
385 C	0.476	848	201	T	

386 C	**0.601**	849	202	T	
387 U	**0.945**	850	203	T	97
388 G	**1.675**	851	204	A	12
389 A	**1.941**	852	205	A	
390 U	**0.334**	853	206	C	
391 G	**0.572**	854	207	T	
392 C	**0.357**	855	208	A	
393 A	**1.703**	856	209	A	11
394 G	**1.856**	857	210	G	2
395 C	**1.044**	858	211	C	
396 C	**0.136**	859	212	T	1
397 A	**1.863**	860	213	A	
398 U	**0.74**	861	214	T	1
399 G	**0.805**	862	215	A	6
400 C	**0.844**	863	216	C	
401 C	**0.769**	864	217	T	
402 G	**0.988**	865	218	A	
403 C	**0.93**	866	219	A	3
	-	867	220	C	6
	-	868	221	C	16
	-	869	222	C	22
	-	870	223	C	28
	-	871	224	A	
404 G	**0.727**				
405 U	**1.24**				
406 G	**0.585**				
407 U	**-0.579**				
408 A	**-0.171**				
409 U	**-0.394**				
410 G	**0.504**				
411 A	**0.489**				
412 A	**-0.342**				
413 G	**0.525**				
414 A	**0.465**				
415 A	**-0.014**				
416 G	**0.351**				
417 G	**0.203**				
418 C	**-0.249**				
419 C	**-0.038**				
420 U	**0.147**				
421 U	**0.011**				
422 C	**-0.296**				
423 G	**0.204**				

424 G	**0.464**
425 G	**-0.218**
426 U	**0.225**
427 U	**0.046**
428 G	**0.513**
429 U	**0.536**
430 A	**0.463**
431 A	**0.566**
432 A	**0.555**
433 G	**-0.391**
434 U	**-0.185**
435 A	**-0.526**
436 C	**0.593**
437 U	**0.503**
438 U	**-0.043**
439 U	**0.545**
440 C	**-0.342**
441 A	**-0.488**
442 G	**-0.64**
443 C	**-0.622**
444 G	**-0.643**
445 G	**-0.107**
446 G	**-0.264**
447 G	**0.187**
448 A	**0.405**
449 G	**-0.572**
450 G	**0.259**
451 A	**0.377**
452 A	**-0.086**
453 G	**-0.802**
454 G	**-0.97**
455 G	**-0.91**
456 A	**-0.938**
457 G	**-0.933**
458 U	**-0.93**
459 A	**-0.921**
460 A	**-0.879**
461 A	**-0.819**
462 G	**-0.797**
463 U	**-0.77**
464 U	**-0.708**
465 A	**-0.555**
466 A	**-0.267**

467 U	**-0.636**			
468 A	**-0.543**			
469 C	**-0.849**			
470 C	**-0.769**			
471 U	**-0.877**			
472 U	**-0.896**			
473 U	**-0.945**			
474 G	**-0.936**			
475 C	**-0.931**			
476 U	**-0.925**			
477 C	**-0.953**			
478 A	**-0.976**			
479 U	**-0.843**			
480 U	**0.266**			
481 G	**0.461**			
482 A	**0.387**			
483 C	**0.261**			
484 G	**0.077**			
485 U	**-0.061**			
486 U	**0.294**			
487 A	**0.408**			
488 C	**-0.277**			
489 C	**-0.142**			
490 C	**-0.601**			
491 G	**-0.638**			
492 C	**-0.678**			
493 A	**-0.575**			
494 G	**-0.144**			
495 A	**0.526**			
496 A	**0.062**			
497 G	**-0.491**			
498 A	**0.765**			
499 A	**1.276**			
500 G	**1.314**	**872**	**225**	G
501 C	**0.606**	**873**	**226**	G
502 A	**0.301**	**874**	**227**	G
503 C	**0.374**	**875**	**228**	T
504 C	**0.821**	**876**	**229**	T
505 G	**1.91**	**877**	**230**	G
506 G	**1.819**	**878**	**231**	G
507 C	**1.513**	**879**	**232**	T
508 U	**0.78**	**880**	**233**	C
509 A	**1.932**	**881**	**234**	A

510 A	1.277	882	235	A	
511 C	0.808	883	236	T	
512 U	1.149	884	237	T	
513 C	0.369	885	238	T	
514 C	0.81	886	239	C	
515 G	1.951	887	240	G	
516 U	1.964	888	241	T	
517 G	1.947	889	242	G	
518 C	1.875	890	243	C	
519 C	1.971	891	244	C	
520 A	1.966	892	245	A	
521 G	1.964	893	246	G	
522 C	1.946	894	247	C	
523 A	1.379	895	248	C	2
524 G	1.486	896	249	A	20
525 C	1.857	897	250	C	
526 C	1.879	898	251	C	
527 G	1.928	899	252	G	
528 C	1.957	900	253	C	
529 G	1.928	901	254	G	
530 G	1.931	902	255	G	
531 U	1.896	903	256	T	
532 A	1.293	904	257	C	
533 A	1.975	905	258	A	
534 U	1.306	906	259	C	1
535 A	1.369	907	260	A	
536 C	1.96	908	261	C	
537 G	0.804	909	262	G	
538 G	0.399	910	263	A	
539 A	0.804	911	264	T	
540 G	0.435	912	265	T	
541 G	0.878	913	266	A	
542 G	0.356	914	267	A	
543 U	0.306	915	268	C	
544 G	0.619	916	269	C	
545 C	1.186	917	270	C	
546 A	0.413	918	271	A	
547 A	1.892	919	272	A	
548 G	1.523	920	273	G	
549 C	1.347	921	274	T	94
550 G	1.366	922	275	C	2
551 U	1.247	923	276	A	
552 U	1.088	924	277	A	

553 A	0.21	925	278	T	
554 A	0.484	926	279	A	
555 U	0.547	927	280	G	
556 C	0.964	928	281	A	15
557 G	0.514	929	282	A	
558 G	0.383				
559 A	0.797				
560 A	0.06				
561 U	1.101				
562 U	0.643				
563 A	0.921				
564 C	0.703	930	283	G	424
565 U	1.38	931	284	C	1
566 G	1.388	932	285	C	
567 G	1.33	933	286	G	
568 G	1.376	934	287	G	1
569 C	1.023	935	288	C	1
570 G	1.029	936	289	G	
571 U	1.92	937	290	T	
572 A	1.836	938	291	A	
573 A	1.817	939	292	A	
574 A	1.938	940	293	A	
575 G	1.291	941	294	G	1
576 C	0.342	942	295	A	16
577 G	1.8	943	296	G	
578 C	0.677	944	297	T	
579 A	0.224	945	298	G	
580 C	0.861	946	299	T	
581 G	1.169	947	300	T	
582 C	0.793	948	301	T	
583 A	1.479	949	302	T	
584 G	1.247	950	303	A	
585 G	0.629	951	304	G	101
586 C	0.546	952	305	A	
	-	953	306	T	2
	-	954	307	C	6
	-	955	308	A	185
	-	956	309	C	23
	-	957	310	C	18
	-	958	311	C	6
	-	959	312	C	21
	-	960	313	C	1
	-	961	314	T	234

	-	962	315	C	4
	-	963	316	C	
	-	964	317	C	3
	-	965	318	C	3
	-	966	319	A	3
	-	967	320	A	
587 G	0.889				
588 G	0.53				
589 U	-0.567				
590 U	-0.322				
591 U	-0.577				
592 G	-0.703				
593 U	-0.481				
594 U	-0.246				
595 A	-0.062				
596 A	-0.017				
597 G	0.515				
598 U	0.231				
599 C	-0.191				
600 A	-0.701				
601 G	-0.274				
602 A	-0.747				
603 U	-0.579				
604 G	0.335				
605 U	0.428				
606 G	-0.06				
607 A	0.398				
608 A	0.573				
609 A	0.574				
610 U	-0.448				
611 C	-0.326				
612 C	-0.095				
613 C	-0.31				
614 C	-0.78				
615 G	-0.567				
616 G	-0.24				
617 G	0.502				
618 C	0.495				
619 U	0.46				
620 C	-0.183				
621 A	0.567				
622 A	0.501				
623 C	0.52				
624 C	-0.306				
625 U	-0.72				

626 G	**-0.732**			
627 G	**-0.234**			
628 G	**-0.055**			
629 A	**-0.456**			
630 A	**-0.581**			
631 C	**-0.717**			
632 U	**-0.298**			
633 G	**0.084**			
634 C	**0.3**			
635 A	**-0.672**			
636 U	**-0.485**			
637 C	**-0.313**			
638 U	**-0.681**			
639 G	**-0.13**			
640 A	**0.127**			
641 U	**-0.332**			
642 A	**0.566**			
643 C	**0.55**			
644 U	**-0.025**			
645 G	**0.014**			
646 G	**-0.621**			
647 C	**-0.784**			
648 A	**-0.631**			
649 A	**-0.46**			
650 G	**-0.577**			
651 C	**0.539**			
652 U	**0.58**			
653 U	**-0.445**			
654 G	**0.581**			
655 A	**0.698**			
656 G	**0.862**			
657 U	**0.411**			
658 C	**-0.128**			
659 U	**0.35**			
660 C	**0.105**			
661 G	**0.654**			
662 U	**0.07**			
663 A	**0.798**			
664 G	**1.187**			
665 A	**0.688**			
666 G	**1.156**			
667 G	**1.256**	**968**	**321**	T
668 G	**0.202**	**969**	**322**	A

669 G	0.112	970	323	A	
670 G	0.812	971	324	A	1
671 G	0.306	972	325	G	
672 U	0.757	973	326	C	
673 A	0.445	974	327	T	
674 G	1.446	975	328	A	
675 A	1.257	976	329	A	
676 A	1.834	977	330	A	
677 U	1.118	978	331	A	6
678 U	1.355	979	332	C	10
679 C	0.537	980	333	T	83
680 C	0.843	981	334	C	1
681 A	0.083	982	335	A	2
682 G	0.144	983	336	C	5
683 G	0.771	984	337	C	1
684 U	0.945	985	338	T	
685 G	0.846	986	339	G	2
686 U	0.396				
687 A	1.709	987	340	A	
688 G	1.663	988	341	G	13
689 C	0.594	989	342	T	
690 G	1.055	990	343	T	12
691 G	1.863	991	344	G	
692 U	1.918	992	345	T	
693 G	1.238	993	346	A	1
694 A	1.508	994	347	A	1
695 A	1.569	995	348	A	
696 A	1.971	996	349	A	1
697 U	1.406	997	350	A	
698 G	0.617	998	351	A	2
699 C	1.612	999	352	C	
	-	1000	353	T	3
	-	1001	354	C	
	-	1002	355	C	9
700 G	0.529				
701 U	0.704				
702 A	0.561				
703 G	0.884				
704 A	1.141				
705 G	0.41				
706 A	0.87	1003	356	A	
707 U	0.938	1004	357	G	
708 C	0.116	1005	358	T	57

709 U	0.079	1006	359	T	
710 G	0.937	1007	360	G	31
711 G	0.582	1008	361	A	5
712 A	1.266	1009	362	C	11
713 G	0.705	1010	363	A	
714 G	1.413	1011	364	C	
715 A	1.784	1012	365	A	
716 A	1.116	1013	366	A	1
717 U	0.286	1014	367	A	2
	-	1015	368	A	
	-	1016	369	T	
	-	1017	370	A	
	-	1018	371	G	1667
	-	1019	372	A	3
718 A	0.913				
719 C	0.839				
720 C	1.404				
721 G	0.275				
722 G	0.688	1020	373	C	
723 U	0.672	1021	374	T	
724 G	0.589				
725 G	1.347	1022	375	A	
726 C	1.679	1023	376	C	
727 G	1.655	1024	377	G	
728 A	1.779	1025	378	A	
729 A	1.901	1026	379	A	
730 G	0.992	1027	380	A	5
731 G	1.651	1028	381	G	
732 C	1.422	1029	382	T	
733 G	0.802	1030	383	G	
734 G	0.329	1031	384	G	
735 C	0.517	1032	385	C	
736 C	0.815	1033	386	T	
737 C	0.113	1034	387	T	
738 C	0.204	1035	388	T	
739 C	1.26	1036	389	A	
	-	1037	390	A	
	-	1038	391	C	2
	-	1039	392	A	
	-	1040	393	T	29
	-	1041	394	A	138
740 U	0.742				
741 G	0.617				
742 G	0.998				

743 A	-0.043				
744 C	0.224				
745 G	-0.004				
746 A	0.091				
747 A	-0.09				
748 G	-0.055				
749 A	0.481				
750 C	0.534				
751 U	0.632				
752 G	0.65				
753 A	0.615				
754 C	0.7				
755 G	0.736	1042	395	T	
756 C	0.808	1043	396	C	
757 U	1.715	1044	397	T	1
	-	1045	398	G	
	-	1046	399	A	
	-	1047	400	A	6
	-	1048	401	C	736
758 C	0.519				
759 A	1.557				
760 G	0.493				
761 G	0.975	1049	402	A	
762 U	0.149	1050	403	C	
763 G	0.655	1051	404	A	
764 C	1.791	1052	405	C	
765 G	1.707	1053	406	A	3
766 A	1.91	1054	407	A	
767 A	1.551	1055	408	T	1
768 A	1.743	1056	409	A	
769 G	1.827	1057	410	G	
770 C	1.254	1058	411	C	
771 G	0.499	1059	412	T	
772 U	0.714	1060	413	A	
773 G	1.504	1061	414	A	1
774 G	1.725	1062	415	G	
775 G	1.567	1063	416	A	2
776 G	0.86	1064	417	C	
777 A	1.406	1065	418	C	
778 G	0.794	1066	419	C	
779 C	1.38	1067	420	A	
780 A	1.012	1068	421	A	
781 A	1.949	1069	422	A	

782 A	1.405	1070	423	C	
783 C	0.643	1071	424	T	
784 A	1.022	1072	425	G	
785 G	1.231	1073	426	G	
786 G	1.942	1074	427	G	
787 A	1.864	1075	428	A	
788 U	1.938	1076	429	T	
789 U	1.461	1077	430	T	
790 A	1.91	1078	431	A	
791 G	1.947	1079	432	G	
792 A	1.925	1080	433	A	
793 U	1.794	1081	434	T	1
794 A	1.935	1082	435	A	
795 C	1.943	1083	436	C	
796 C	1.951	1084	437	C	
797 C	1.249	1085	438	C	
798 U	1.014	1086	439	C	
799 G	0.59	1087	440	A	
800 G	1.265	1088	441	C	
801 U	1.935	1089	442	T	
802 A	1.927	1090	443	A	
803 G	1.364	1091	444	T	
804 U	1.36	1092	445	G	
805 C	1.686	1093	446	C	
806 C	1.234	1094	447	T	1
807 A	0.695	1095	448	T	20
808 C	0.481	1096	449	A	
809 G	1.251	1097	450	G	
810 C	1.81	1098	451	C	
811 C	1.011	1099	452	C	
812 G	0.713	1100	453	C	
813 U	1.801	1101	454	T	
814 A	1.83	1102	455	A	
815 A	1.881	1103	456	A	
816 A	1.861	1104	457	A	
817 C	1.571	1105	458	C	
818 G	0.864	1106	459	C	9
819 A	0.689	1107	460	T	109
820 U	1.437	1108	461	C	
821 G	1.415	1109	462	A	
822 U	-0.025	1110	463	A	
823 C	-0.165	1111	464	C	
824 G	-0.141	1112	465	A	

825 A	0.519	1113	466	G	
826 C	1.023	1114	467	T	
827 U	1.224	1115	468	T	
	-	1116	469	A	2
	-	1117	470	A	14
	-	1118	471	A	3
	-	1119	472	T	75
	-	1120	473	C	15
	-	1121	474	A	
	-	1122	475	A	
	-	1123	476	C	
	-	1124	477	A	
	-	1125	478	A	
828 U	0.449				
829 G	0.615				
830 G	-0.205				
831 A	-0.169				
832 G	0.383				
833 G	0.441				
834 U	-0.288				
835 U	0.02				
836 G	0.505				
837 U	-0.415				
838 G	-0.554				
839 C	-0.652				
840 C	-0.872				
841 C	-0.874				
842 U	-0.085				
843 U	-0.378				
844 G	-0.624				
845 A	-0.531				
846 G	-0.772				
847 G	-0.709				
848 C	-0.66				
849 G	-0.489				
850 U	0.094				
851 G	-0.227				
852 G	-0.419				
853 C	0.333				
854 U	0.123				
855 U	-0.125				
856 C	-0.196				
857 C	0.34				
858 G	0.162				

859 G	-0.434				
860 A	0.559				
861 G	0.649				
862 C	0.232				
863 U	0.529				
864 A	1.511				
865 A	1.89				
866 C	1.012				
867 G	0.463				
868 C	0.829				
869 G	0.087				
870 U	-0.031				
871 U	0.31				
872 A	1.368				
873 A	1.085	1126	479	A	
874 G	1.004	1127	480	A	
875 U	0.648	1128	481	C	
876 C	0.016	1129	482	T	
877 G	-0.018	1130	483	G	
878 A	0.026	1131	484	C	
879 C	1.137	1132	485	T	1
880 C	1.261	1133	486	C	1
881 G	1.023	1134	487	G	
882 C	1.533	1135	488	C	
883 C	1.311	1136	489	C	
884 U	0.801	1137	490	A	
885 G	1.846	1138	491	G	
886 G	1.377	1139	492	A	
887 G	1.746	1140	493	A	
888 G	0.892	1141	494	C	
889 A	1.95	1142	495	A	
890 G	1.274	1143	496	C	
891 U	1.951	1144	497	T	
892 A	1.952	1145	498	A	
893 C	1.196	1146	499	C	
894 G	1.454	1147	500	G	
895 G	0.518	1148	501	A	2
896 C	0.337	1149	502	G	
897 C	1.568	1150	503	C	
898 G	1.102	1151	504	C	
899 C	1.398	1152	505	A	
900 A	1.433	1153	506	C	
901 A	1.696	1154	507	A	

902 G	1.559	1155	508	G	
903 G	0.314	1156	509	C	
904 U	0.351	1157	510	T	
905 U	1.76	1158	511	T	
906 A	0.896	1159	512	A	
907 A	1.943	1160	513	A	
908 A	1.971	1161	514	A	1
909 A	1.957	1162	515	A	
910 C	1.834	1163	516	C	
911 U	1.7	1164	517	T	
912 C	1.193	1165	518	C	
913 A	1.724	1166	519	A	1
914 A	1.961	1167	520	A	
915 A	1.931	1168	521	A	
916 U	1.411	1169	522	G	
917 G	1.59	1170	523	G	
918 A	1.897	1171	524	A	
919 A	1.277	1172	525	C	
920 U	1.834	1173	526	C	1
921 U	1.775	1174	527	T	
922 G	1.944	1175	528	G	
923 A	1.309	1176	529	G	
924 C	1.94	1177	530	C	
925 G	1.901	1178	531	G	
926 G	1.971	1179	532	G	
927 G	0.763	1180	533	T	
928 G	1.14	1181	534	G	
929 G	0.601	1182	535	C	
930 C	0.675	1183	536	T	1
931 C	0.765	1184	537	T	
932 C	1.107	1185	538	C	8
933 G	0.956	1186	539	A	
934 C	1.071				
	-	1187	540	T	18
	-	1188	541	A	
	-	1189	542	T	738
935 A	1.308	1190	543	C	
936 C	1.798	1191	544	C	
937 A	1.092	1192	545	C	4
938 A	1.058	1193	546	T	51
939 G	1.204	1194	547	C	
940 C	0.643	1195	548	T	
941 G	0.687	1196	549	A	

942 G	1.829	1197	550	G	
943 U	0.682	1198	551	A	1
944 G	1.951	1199	552	G	
945 G	1.852	1200	553	G	
946 A	1.897	1201	554	A	
947 G	1.425	1202	555	G	
948 C	1.366	1203	556	C	
949 A	0.845	1204	557	C	
950 U	1.915	1205	558	T	
951 G	1.926	1206	559	G	
952 U	1.175	1207	560	T	
953 G	1.1	1208	561	T	
954 G	1.188	1209	562	C	
955 U	0.732	1210	563	T	
	-	1211	564	G	26
956 U	1.259				
957 U	1.713	1212	565	T	
958 A	1.941	1213	566	A	
959 A	1.045				
960 U	1.354	1214	567	A	
961 U	1.725	1215	568	T	
962 C	1.146	1216	569	C	
963 G	1.959	1217	570	G	
964 A	1.965	1218	571	A	
965 U	0.393	1219	572	T	
966 G	0.623	1220	573	A	
967 C	0.98	1221	574	A	
968 A	1.019	1222	575	A	6
969 A	1.316	1223	576	C	
970 C	1.845	1224	577	C	
971 G	0.722	1225	578	C	
972 C	1.957	1226	579	C	
973 G	1.708	1227	580	G	1
974 A	0.588	1228	581	A	
975 A	0.644	1229	582	T	
976 G	0.813	1230	583	C	
977 A	1.421	1231	584	A	
978 A	1.933	1232	585	A	
979 C	1.214	1233	586	C	
980 C	1.904	1234	587	C	
981 U	1.739	1235	588	T	2
982 U	1.042	1236	589	C	
983 A	1.944	1237	590	A	

984 C	1.965	1238	591	C	
985 C	1.856	1239	592	C	
986 U	0.632	1240	593	A	1
987 G	0.446	1241	594	C	1
988 G	0.554	1242	595	C	
989 U	0.185	1243	596	T	351
990 C	1.1	1244	597	C	
991 U	0.914	1245	598	T	
992 U	0.571	1246	599	T	1
993 G	0.786	1247	600	G	
	-	1248	601	C	
	-	1249	602	T	
	-	1250	603	C	
994 A	0.284				
995 C	0.253				
996 A	0.186				
997 U	0.214				
998 C	-0.734				
999 C	-0.785				
1000 A	-0.781				
1001 C	-0.887				
1002 G	-0.869				
1003 G	-0.345				
1004 A	-0.338				
1005 A	-0.601				
1006 G	-0.926				
1007 U	-0.89				
1008 U	-0.825				
1009 U	-0.79				
1010 U	-0.815				
1011 C	-0.617				
1012 A	-0.427				
1013 G	0.052				
1014 A	0.467				
1015 G	-0.267				
1016 A	0.128				
1017 U	-0.413				
1018 G	-0.618				
1019 A	-0.831				
1020 G	-0.8				
1021 A	-0.795				
1022 A	-0.874				
1023 U	-0.922				

1024 G	-0.7				
1025 U	-0.541				
1026 G	-0.721				
1027 C	-0.543				
1028 C	-0.291				
1029 U	-0.434				
1030 U	-0.146				
1031 C	-0.729				
1032 G	-0.477				
1033 G	-0.198				
1034 G	-0.474				
1035 A	-0.632				
1036 A	-0.601				
1037 C	-0.581				
1038 C	-0.896				
1039 G	-0.86				
1040 U	-0.754				
1041 G	-0.733				
1042 A	-0.586				
1043 G	-0.762				
1044 A	0.083				
1045 C	0.616				
1046 A	1.238	1251	604	A	
1047 G	1.832	1252	605	G	
1048 G	1.181	1253	606	C	
1049 U	1.183	1254	607	C	
1050 G	1.316	1255	608	T	1
1051 C	0.482	1256	609	A	
1052 U	1.784	1257	610	T	
1053 G	1.346	1258	611	A	
1054 C	1.363	1259	612	T	
1055 A	1.958	1260	613	A	
1056 U	1.309	1261	614	C	
1057 G	1.322	1262	615	C	
1058 G	1.714	1263	616	G	1
1059 C	1.168	1264	617	C	
1060 U	1.036	1265	618	C	
1061 G	1.639	1266	619	A	1
1062 U	1.825	1267	620	T	
1063 C	1.348	1268	621	C	
1064 G	0.987	1269	622	T	
1065 U	1.574	1270	623	T	
1066 C	1.253	1271	624	C	

1067 A	1.89	1272	625	A	
1068 G	1.836	1273	626	G	
1069 C	1.221	1274	627	C	
1070 U	1.409	1275	628	A	1
1071 C	0.808	1276	629	A	
1072 G	1.323	1277	630	A	
1073 U	1.361	1278	631	C	
1074 G	1.156	1279	632	C	
1075 U	0.596	1280	633	C	
1076 U	0.64	1281	634	T	
1077 G	1.224	1282	635	G	4
	-	1283	636	A	
1078 U	1.337	1284	637	T	15
1079 G	1.308	1285	638	G	
1080 A	1.645	1286	639	A	
1081 A	0.713	1287	640	A	1
1082 A	0.563	1288	641	G	
1083 U	1.114	1289	642	G	1
	-	1290	643	C	6
	-	1291	644	T	6
	-	1292	645	A	2
	-	1293	646	C	
	-	1294	647	A	
	-	1295	648	A	
1084 G	0.925				
1085 U	1.05				
1086 U	0.349				
1087 G	0.089				
1088 G	0.866				
1089 G	0.846				
1090 U	1.219				
1091 U	1.161				
1092 A	1.25				
1093 A	1.438				
1094 G	0.616				
1095 U	1.008				
1096 C	0.836				
1097 C	0.834				
1098 C	0.157				
1099 G	0.219				
1100 C	0.627				
1101 A	1.889	1296	649	A	
1102 A	1.358	1297	650	G	

1103 C	1.292	1298	651	T	
1104 G	1.424	1299	652	A	6
1105 A	1.684	1300	653	A	
1106 G	1.314	1301	654	G	
1107 C	1.834	1302	655	C	
1108 G	1.209	1303	656	G	16
1109 C	0.878	1304	657	C	
1110 A	1.194	1305	658	A	
1111 A	1.892	1306	659	A	1
1112 C	1.188	1307	660	G	
1113 C	1.107	1308	661	T	1
1114 C	0.756	1309	662	A	3
1115 U	0.659	1310	663	C	22
	-	1311	664	C	
	-	1312	665	C	
	-	1313	666	A	4
	-	1314	667	C	
1116 U	-0.195				
1117 A	0.042				
1118 U	-0.182				
1119 C	-0.316				
1120 C	-0.689				
1121 U	-0.16				
1122 U	-0.405				
1123 U	-0.213				
1124 G	0.132				
1125 U	0.505				
1126 U	0.415				
1127 G	0.098				
1128 C	0.362				
1129 C	-0.031				
1130 A	0.336				
1131 G	-0.532				
1132 C	0.282				
1133 G	-0.32				
1134 G	-0.909				
1135 U	-0.021				
1136 C	-0.707				
1137 C	-0.403				
1138 G	-0.561				
1139 G	-0.406				
1140 C	-0.883				
1141 C	-0.317				

1142 G	**0.276**
1143 G	**0.013**
1144 G	**0.365**
1145 A	**-0.684**
1146 A	**0.37**
1147 C	**0.411**
1148 U	**0.281**
1149 C	**0.154**
1150 A	**-0.336**
1151 A	**-0.43**
1152 A	**-0.371**
1153 G	**-0.682**
1154 G	**-0.06**
1155 A	**-0.174**
1156 G	**0.515**
1157 A	**1.005**
1158 C	**0.969**
1159 U	**0.487**
1160 G	**0.539**
1161 C	**0.459**
1162 C	**0.428**
1163 A	**0.036**
1164 G	**0.304**
1165 U	**-0.258**
1166 G	**0.566**
1167 A	**-0.23**
1168 U	**-0.032**
1169 A	**0.381**
1170 A	**0.851**
1171 A	**-0.33**
1172 C	**-0.076**
1173 U	**0.025**
1174 G	**0.429**
1175 G	**0.465**
1176 A	**0.691**
1177 G	**1.003**
1178 G	**1.025**
1179 A	**1.252**
1180 A	**1.189**
1181 G	**0.913**
1182 G	**0.323**
1183 U	**0.403**
1184 G	**0.292**

1185 G	0.822	1315	668	G	1
1186 G	0.725	1316	669	T	
1187 G	1.121	1317	670	A	
1188 A	1.092	1318	671	A	
1189 U	0.486	1319	672	A	
1190 G	0.726	1320	673	G	
1191 A	1.947	1321	674	A	
1192 C	1.001	1322	675	C	
1193 G	1.316	1323	676	G	
1194 U	1.107	1324	677	T	
1195 C	1.56	1325	678	T	
1196 A	1.911	1326	679	A	
1197 A	1.056	1327	680	G	
1198 G	1.18	1328	681	G	
1199 U	1.91	1329	682	T	
1200 C	1.939	1330	683	C	
1201 A	0.684	1331	684	A	2
1202 U	0.673	1332	685	A	
1203 C	0.638	1333	686	G	
1204 A	1.362	1334	687	G	
1205 U	1.792	1335	688	T	
1206 G	1.746	1336	689	G	
1207 G	0.553	1337	690	T	
1208 C	1.258	1338	691	A	
1209 C	1.192	1339	692	G	
1210 C	1.21	1340	693	C	
1211 U	1.04	1341	694	C	7
1212 U	0.91	1342	695	C	15
1213 A	1.942	1343	696	A	
1214 C	0.998	1344	697	T	
1215 G	1.409	1345	698	G	1
1216 A	0.138	1346	699	A	3
1217 C	0.247	1347	700	G	
1218 C	0.436	1348	701	G	1
1219 A	0.609	1349	702	T	
1220 G	1.833	1350	703	G	
1221 G	1.96	1351	704	G	
	-	1352	705	C	2
	-	1353	706	A	
1222 G	1.068				
1223 C	1.057				
1224 U	0.568				
1225 A	0.767	1354	707	A	

1226 C	1.261	1355	708	G	
1227 A	1.729	1356	709	A	
1228 C	1.14	1357	710	A	
1229 A	1.192	1358	711	A	
1230 C	1.355	1359	712	T	
1231 G	1.428	1360	713	G	
1232 U	0.795	1361	714	G	
1233 G	1.447	1362	715	G	
1234 C	1.492	1363	716	C	
1235 U	1.9	1364	717	T	
1236 A	1.936	1365	718	A	
1237 C	1.967	1366	719	C	
1238 A	1.941	1367	720	A	1
1239 A	0.864	1368	721	T	3
1240 U	1.906	1369	722	T	
1241 G	1.204	1370	723	T	
1242 G	0.725	1371	724	T	
1243 C	0.334	1372	725	C	
1244 G	0.002	1373	726	T	
1245 C	0.179	1374	727	A	2
	-	1375	728	C	6
	-	1376	729	C	
	-	1377	730	C	4
1246 A	-0.214				
1247 U	-0.037				
1248 A	0.496				
1249 C	0.494				
1250 A	0.53				
1251 A	-0.172				
1252 A	-0.297				
1253 G	0.266				
1254 A	-0.324				
1255 G	0.491				
1256 A	-0.618				
1257 A	-0.613				
1258 G	0.271				
1259 C	0.401				
1260 G	-0.694				
1261 A	0.356				
1262 C	-0.817				
1263 C	-0.728				
1264 U	-0.428				
1265 C	-0.539				

1266 G	0.278				
1267 C	-0.155				
1268 G	-0.007				
1269 A	0.459				
1270 G	-0.409				
1271 A	-0.378				
1272 G	-0.558				
1273 C	-0.47				
1274 A	-0.327				
1275 A	0.132				
1276 G	0.417				
1277 C	0.275				
1278 G	-0.816				
1279 G	0.307				
1280 A	0.504				
1281 C	0.01				
1282 C	0.439				
1283 U	-0.44				
1284 C	-0.068				
1285 A	-0.543				
1286 U	-0.889				
1287 A	0.495				
1288 A	0.522				
1289 A	0.513				
1290 G	-0.396				
1291 U	-0.241				
1292 G	0.179	1378	731	C	
1293 C	-0.009	1379	732	A	
1294 G	0.339	1380	733	G	
1295 U	0.819	1381	734	A	
1296 C	1.202	1382	735	A	106
	-	1383	736	A	
	-	1384	737	A	
	-	1385	738	C	
	-	1386	739	T	33
	-	1387	740	A	
1297 G	0.22				
1298 U	0.453				
1299 A	0.737				
1300 G	0.436				
1301 U	1.024				
1302 C	0.293				
1303 C	1.248	1388	741	C	

1304 G	1.694	1389	742	G	2
1305 G	1.441	1390	743	A	
1306 A	1.796	1391	744	T	35
1307 U	1.278	1392	745	A	1
1308 U	0.479	1393	746	G	36
1309 G	1.285	1394	747	C	
1310 G	0.033	1395	748	C	
1311 A	0.33	1396	749	C	4
1312 G	0.626	1397	750	T	
1313 U	0.44	1398	751	T	
1314 C	0.804	1399	752	A	1
1315 U	1.95	1400	753	T	
1316 G	1.928	1401	754	G	
1317 C	0.964	1402	755	A	1
1318 A	1.962	1403	756	A	
1319 A	1.939	1404	757	A	
1320 C	0.747				
1321 U	0.942				
1322 C	0.367				
	-	1405	758	C	5
	-	1406	759	T	59
1323 G	0.813	1407	760	T	
1324 A	0.467	1408	761	A	1
1325 C	0.658	1409	762	A	
1326 U	0.327	1410	763	G	
1327 C	0.02	1411	764	G	
1328 C	1.29	1412	765	G	
1329 A	0.45	1413	766	T	21
1330 U	1.346	1414	767	C	
1331 G	1.334	1415	768	G	16
1332 A	1.969	1416	769	A	
1333 A	1.961	1417	770	A	
1334 G	1.267	1418	771	G	
1335 U	0.399	1419	772	G	
1336 C	0.468	1420	773	T	36
1337 G	1.97	1421	774	G	
1338 G	1.944	1422	775	G	
1339 A	1.972	1423	776	A	
1340 A	0.736	1424	777	T	
1341 U	1.964	1425	778	T	
1342 C	0.76	1426	779	T	
1343 G	0.686	1427	780	A	
1344 C	1.254	1428	781	G	

1345 U	1.456	1429	782	C	
1346 A	1.663	1430	783	A	
1347 G	1.975	1431	784	G	
1348 U	1.979	1432	785	T	
1349 A	1.965	1433	786	A	
1350 A	1.761	1434	787	A	
1351 U	0.753	1435	788	A	
1352 C	1.141	1436	789	C	
1353 G	1.099	1437	790	T	
1354 U	0.617	1438	791	A	16721
1355 G	0.738	1439	792	A	
1356 G	0.64	1440	793	G	2
1357 A	1.014	1441	794	A	
1358 U	1.247	1442	795	G	166
1359 C	1.319	1443	796	T	1
1360 A	1.92	1444	797	A	
1361 G	1.033	1445	798	G	
1362 A	0.874	1446	799	A	
1363 A	1.276	1447	800	G	
1364 U	0.389	1448	801	T	
1365 G	0.882	1449	802	G	
1366 C	0.621	1450	803	C	
1367 C	0.758	1451	804	T	
1368 A	0.687	1452	805	T	13
1369 C	1.119	1453	806	A	50
1370 G	1.162	1454	807	G	
1371 G	0.846	1455	808	T	
1372 U	1.716	1456	809	T	
1373 G	1.634	1457	810	G	
1374 A	1.923	1458	811	A	
1375 A	1.418	1459	812	A	
1376 U	1.309	1460	813	C	
1377 A	1.052	1461	814	A	7
1378 C	1.188	1462	815	G	111
1379 G	1.719	1463	816	G	
1380 U	1.339	1464	817	G	
1381 U	0.959	1465	818	C	2
1382 C	1.459	1466	819	C	1
1383 C	1.271	1467	820	C	
1384 C	1.016	1468	821	T	
1385 G	1.582	1469	822	G	
1386 G	0.84	1470	823	A	
1387 G	0.808	1471	824	A	

1388 C	0.678	1472	825	G	2
1389 C	1.133	1473	826	C	3
1390 U	1.246	1474	827	G	
1391 U	1.453	1475	828	C	
1392 G	1.963	1476	829	G	
1393 U	1.562	1477	830	T	
1394 A	1.971	1478	831	A	
1395 C	1.986	1479	832	C	
1396 A	1.799	1480	833	A	
1397 C	1.712	1481	834	C	
1398 A	1.966	1482	835	A	
1399 C	1.874	1483	836	C	
1400 C	1.797	1484	837	C	
1401 G	1.885	1485	838	G	
1402 C	1.94	1486	839	C	
1403 C	1.883	1487	840	C	
1404 C	1.953	1488	841	C	
1405 G	1.941	1489	842	G	
1406 U	1.957	1490	843	T	
1407 C	1.94	1491	844	C	
1408 A	1.289	1492	845	A	
1409 C	1.346	1493	846	C	
1410 A	0.24	1494	847	C	3
1411 C	1.016	1495	848	C	1
1412 C	1.245	1496	849	T	
1413 A	0.778	1497	850	C	
1414 U	0.487	1498	851	C	1
1415 G	0.669	1499	852	T	
1416 G	0.305	1500	853	C	
	-	1501	854	A	
	-	1502	855	A	
	-	1503	856	G	46
1417 G	0.439				
1418 A	1.159				
1419 G	0.371	1504	857	T	
1420 U	0.115	1505	858	A	
1421 G	-0.465	1506	859	T	
1422 G	0.162	1507	860	A	
	-	1508	861	C	13
	-	1509	862	T	
	-	1510	863	T	
	-	1511	864	C	1
	-	1512	865	A	

	-	1513	866	A	
	-	1514	867	A	
	-	1515	868	G	
	-	1516	869	G	
	-	1517	870	A	2
	-	1518	871	C	
	-	1519	872	A	
	-	1520	873	T	13
	-	1521	874	T	
	-	1522	875	T	
	-	1523	876	A	
	-	1524	877	A	11
	-	1525	878	C	
	-	1526	879	T	
	-	1527	880	A	
	-	1528	881	A	
	-	1529	882	A	
	-	1530	883	A	5
	-	1531	884	C	7
	-	1532	885	C	
	-	1533	886	C	
	-	1534	887	C	
	-	1535	888	T	
	-	1536	889	A	11
	-	1537	890	C	2
	-	1538	891	G	
	-	1539	892	C	1
	-	1540	893	A	
	-	1541	894	T	24
	-	1542	895	T	2
1423 G	0.171				
1424 U	-0.286				
1425 U	-0.298				
1426 G	-0.393				
1427 C	0.08				
1428 A	-0.137				
1429 A	0.097				
1430 A	0.038				
1431 A	-0.175				
1432 G	0.615				
1433 A	1.049				
1434 A	0.453				
1435 G	0.533				

1436 U	**-0.281**			
1437 A	**-0.301**			
1438 G	**-0.423**			
1439 G	**-0.391**			
1440 U	**0.028**			
1441 A	**-0.163**			
1442 G	**-0.38**			
1443 C	**-0.671**			
1444 U	**-0.295**			
1445 U	**-0.277**			
1446 A	**0.033**			
1447 A	**-0.072**			
1448 C	**0.027**			
1449 C	**-0.181**			
1450 U	**-0.396**			
1451 U	**-0.148**			
1452 C	**-0.361**			
1453 G	**-0.474**			
1454 G	**-0.032**			
1455 G	**0.014**			
1456 A	**-0.195**			
1457 G	**-0.002**			
1458 G	**-0.005**			
1459 G	**-0.508**			
1460 C	**-0.169**			
1461 G	**0.291**			
1462 C	**-0.373**			
1463 U	**-0.233**			
1464 U	**-0.216**			
1465 A	**-0.484**			
1466 C	**0.287**			
1467 C	**-0.324**			
1468 A	**1.004**			
1469 C	**0.011**			
1470 U	**-0.005**			
1471 U	**0.046**			
1472 U	**0.436**			
1473 G	**-0.254**			
1474 U	**-0.229**			
1475 G	**-0.09**			
1476 A	**-0.433**			
1477 U	**-0.295**			
1478 U	**-0.079**	**1543**	**896**	T

1479 C	-0.554	1544	897	A	
1480 A	-0.218	1545	898	T	
1481 U	-0.24	1546	899	A	1
1482 G	0.686	1547	900	T	
1483 A	1.478	1548	901	A	
1484 C	0.302	1549	902	G	
1485 U	0.512	1550	903	A	
1486 G	0.817	1551	904	G	
1487 G	1.204	1552	905	G	
1488 G	1.142	1553	906	A	
1489 G	0.991	1554	907	G	
1490 U	0.201	1555	908	A	27
1491 G	0.515	1556	909	C	
1492 A	1.917	1557	910	A	
1493 A	1.914	1558	911	A	
1494 G	1.936	1559	912	G	
1495 U	1.945	1560	913	T	
1496 C	1.877	1561	914	C	
1497 G	1.954	1562	915	G	
1498 U	1.827	1563	916	T	
1499 A	1.95	1564	917	A	
1500 A	1.937	1565	918	A	
1501 C	1.864	1566	919	C	
1502 A	1.967	1567	920	A	
1503 A	1.315	1568	921	T	
1504 G	1.808	1569	922	G	
1505 G	1.936	1570	923	G	
1506 U	1.901	1571	924	T	
1507 A	1.098	1572	925	A	
1508 A	0.379	1573	926	A	
1509 C	1.097	1574	927	G	
1510 C	1.147	1575	928	T	
1511 G	1.241	1576	929	G	
1512 U	1.854	1577	930	T	
1513 A	1.756	1578	931	A	3
1514 G	0.575	1579	932	C	
1515 G	0.359	1580	933	T	
1516 G	0.847	1581	934	G	
1517 G	1.854	1582	935	G	
1518 A	1.848	1583	936	A	
1519 A	1.888	1584	937	A	
1520 C	0.216	1585	938	A	2
1521 C	0.824	1586	939	G	

1522 U	**1.717**	**1587**	**940**	T	
1523 G	**1.743**	**1588**	**941**	G	
1524 C	**1.042**	**1589**	**942**	C	
1525 G	**0.769**	**1590**	**943**	A	
1526 G	**0.967**	**1591**	**944**	C	
1527 U	**0.263**	**1592**	**945**	T	
1528 U	**0.913**	**1593**	**946**	T	
1529 G	**1.578**	**1594**	**947**	G	
1530 G	**1.741**	**1595**	**948**	G	
1531 A	**1.423**	**1596**	**949**	A	1
1532 U	**0.874**	**1597**	**950**	C	
1533 C	**0.601**	**1598**	**951**	G	213
1534 A	**1.202**	**1599**	**952**	A	
1535 C	**-0.503**	**1600**	**953**	A	
1536 C	**-0.406**	**1601**	**954**	C	2
1537 U	**-0.302**				
1538 C	**-0.347**				
1539 C	**-0.348**				
1540 U	**-0.281**				
1541 U	**-0.355**				
1542 A	**-0.79**				

* Cannone J.J., Subramanian S., Schnare M.N., Collett J.R., D'Souza L.M., Du Y., Feng B., Lin N., Madabusi L.V., Muller K.M. *et al.* (2002) The comparative RNA web (CRW) site: An online database of comparative sequence and structure information for ribosomal, intron, and other RNAs. *BMC Bioinformatics*, **3**, 2.
